# Photocatalytic Defluorination of Perfluorooctanoic Acid by Twisted Linear Polymer Radicals

**DOI:** 10.1021/jacs.5c17497

**Published:** 2026-05-13

**Authors:** Jiaxi Hu, Yan Guo, Qixin Zhou, Ling Zhang, Haoying Wang, Junshan Li, Bin Liu, Yongfa Zhu

**Affiliations:** † Department of Chemistry, 12442 Tsinghua University, Beijing 100084, China; ‡ Department of Civil Engineering, 25809 The University of Hong Kong, Hong Kong SAR 999077, China; § Department of Materials Science and Engineering, 53025 City University of Hong Kong, Hong Kong SAR 999077, China; ∥ Department of Chemistry, Hong Kong Institute for Clean Energy Center (HKICE), Center of Super-Diamond and Advanced Films (COSDAF), City University of Hong Kong, Hong Kong SAR 999077, China; ⊥ Institute for Advanced Study, 74707 Chengdu University, Chengdu 610106, P. R. China

## Abstract

The exceptional persistence of per- and polyfluoroalkyl substances (PFAS) rooted in inert C–F bonds demands remediation strategies beyond energy-intensive treatments. Here, we report a bithiophene–fluorene–pyridine (BT–Fl–Py) linear polymer that achieves quantitative defluorination of perfluorooctanoic acid (PFOA) under visible-light irradiation. Upon photoexcitation, the polymer undergoes configurational torsional relaxation of the fluorene π-bridge, forming a stable twisted intramolecular charge-transfer state (TICT_1_) that stabilizes photogenerated electrons in a long-lived reducing state. Concurrently, the hydrophobic polymer backbone enriches PFOA, activating C–F bonds and facilitating efficient interfacial electron transfer. The cooperation between conformationally regulated charge separation and interfacial substrate enrichment enables complete PFOA defluorination under mild conditions, establishing a sustainable route for degrading ultrastable PFAS and providing a molecular design principle for developing reducing polymer photocatalysts.

## Graphical abstract

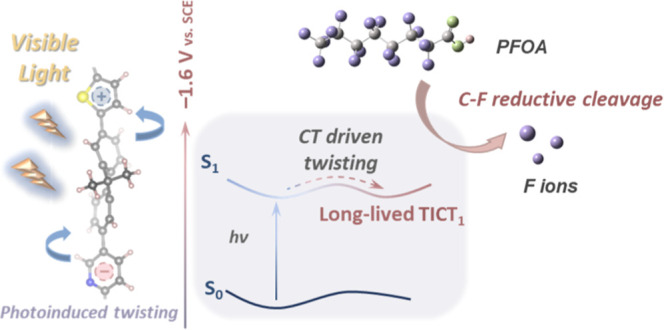

## Introduction

Per- and polyfluoroalkyl substances (PFAS) represent a class of synthetic compounds that have been extensively utilized as surfactants and surface treatment agents since the 1940s.^1,2^ Their widespread production and application have led to global environmental persistence, posing a significant threat to ecosystems^3^ and human health^4^ due to their propensity for long-range transport, high toxicity, and exceptional chemical stability. As a paradigm of this class, perfluorooctanoic acid (PFOA) has been strictly regulated under the Stockholm Convention and recently classified as “carcinogenic to humans” (Group 1) by the International Agency for Research on Cancer (IARC).^5^ The pervasive detection of PFAS in natural water, human serum, and public water supplies^6–8^ underscores the critical needs for effective remediation technologies.

The recalcitrance of PFAS arises from the formidable strength of the carbon–fluorine (C–F) bond (bond dissociation energy ≈485 kJ mol^–1^), which resists conventional degradation processes.^9^ Although adsorption,^10^ filtration,^11^ ion exchange,^12^ and chemical oxidation^13,14^ can sequester PFAS, complete C–F bond cleavage is required to avoid secondary pollution. Existing destructive technologies for near-complete defluorination often demand high-energy inputs (e.g., thermal,^15,16^ plasma,^17^ ultraviolet,^18^ or electrochemical^19,20^ activation) or stoichiometric chemical reagents.^21^ A fundamental limitation of these methods is their inefficacy at the ultratrace concentrations (typically parts-per-trillion) relevant to contaminated water supplies,^22^ where the amphiphilic nature of PFAS complicates both enrichment and degradation: hydrophilic head groups promote aqueous dispersion, while hydrophobic perfluorinated tails adsorb strongly to interfaces.

Heterogeneous photocatalysis provides a promising alternative by utilizing light, rather than fossil energy or chemicals, to drive remediation.^23^ The challenge lies in designing a photocatalyst capable of simultaneously concentrating ambient PFAS and enabling efficient reductive C–F bond cleavage, which remains thermodynamically and kinetically demanding even on catalytic Au electrodes, with an apparent E°' of ca. −1.8 V vs Ag/AgCl and calculated C–F dissociation potentials of −2.06 to −2.52 V vs Ag/AgCl^24^. Although tandem photoexcitation strategies have been developed to access such high reductive driving forces,^25,26^ they often require complex excitation schemes and sacrificial electron donors and are not universally achievable. Single-photon excitation represents a simpler and more sustainable alternative, but its performance is often limited by fast charge recombination. Therefore, a critical yet unmet need is to stabilize long-lived, strongly reducing charge-separated states under single-photon excitation so that efficient defluorination can be achieved without exogenous sacrificial agents. While conventional donor–acceptor (D–A) structures promote charge separation,^27,28^ strong Coulombic attraction typically limits excited-state persistence. We thus reasoned that introducing structural motifs that enable nonadiabatic torsional relaxation could spatially decouple charges and prolong the lifetime of the charge-separated state. This design allows photogenerated electrons to be efficiently utilized for interfacial electron transfer to adsorbed PFOA, rather than being lost through rapid recombination.

To address these challenges, in this work, we report a linear polymer photocatalyst, bithiophene–fluorene–pyridine (BT–Fl–Py), that enables efficient defluorination of PFAS under visible-light irradiation without externally added sacrificial agents. As illustrated in Scheme 1, the polymer structure features an asymmetric fluorene (Fl) π spacer positioned between the electron-donating bithiophene (BT) unit and the electron-accepting pyridine (Py) group. Upon visible-light single-photon excitation, the system undergoes nonadiabatic configurational torsional relaxation along the Fl π-bridge, forming a long-lived, stable twisted intramolecular charge-transfer state (TICT) denoted TICT_1_. This TICT_1_ exhibits a reduction potential of −1.6 V vs SCE and mediates direct interfacial electron transfer to PFOA, driving reductive defluorination. Combined experimental and computational studies further reveal that the hydrophobic polymer backbone enriches PFOA at the interface, while electrostatic interactions facilitate efficient electron transfer to C–F bonds. This single-photon-driven TICT_1_-mediated mechanism enables the efficient and nearly quantitative defluorination of PFOA, representing a significant advance toward sustainable PFAS remediation.

**Scheme 1:**
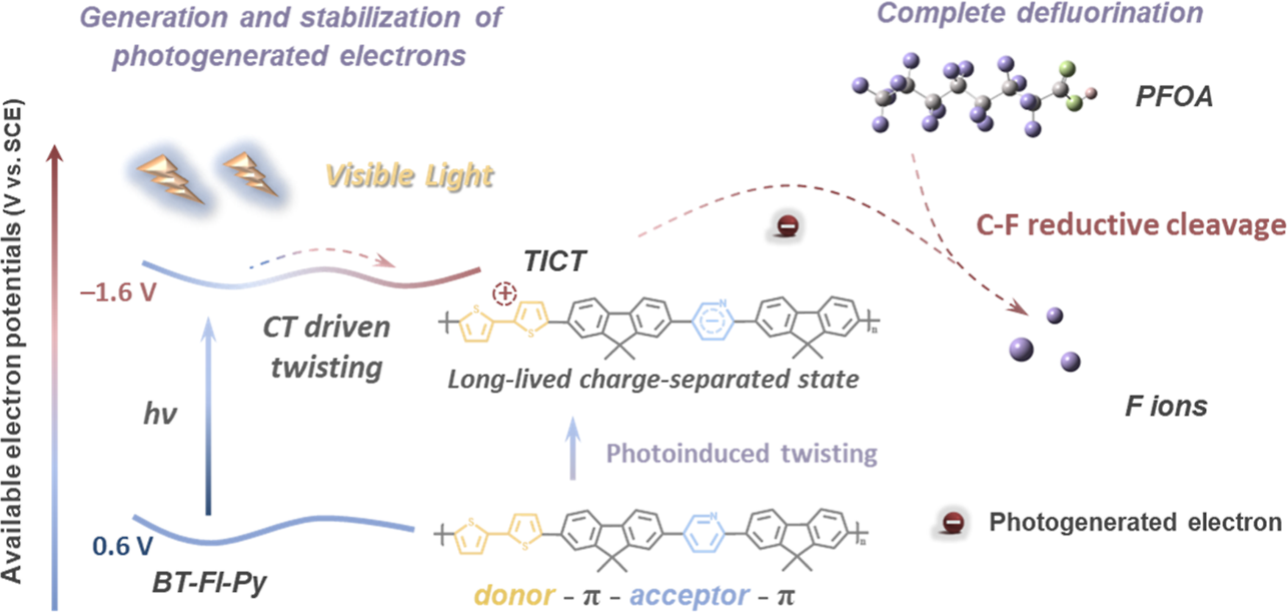
Schematic Illustration Showing the Photoreductive Defluorination of PFOA by Twisted Linear Polymer

## Results and Discussion

### Photoinduced Twisting of the BT–Fl–Py Linear Polymer

The bithiophene–fluorene–pyridine (BT–Fl–Py), along with control polymers bithiophene–fluorene (BT–Fl) and fluorene homopolymer (Fl), were synthesized via the Suzuki cross-coupling polymerization reaction (Figure 1a, Schemes S1–S3). Their chemical structures were characterized by solid-state ^13^C nuclear magnetic resonance (NMR) and Fourier transform infrared (FT-IR) spectroscopy. The ^13^C NMR spectra (Figure 1b) exhibited broad resonances between 110 and 150 ppm, characteristic of aromatic carbons. A distinct peak at ∼144 ppm is assigned to C atoms bound to S atom in the thiophene units,^29^ while signals at ∼120 ppm correspond to the central carbons of the fluorene moiety. The FT-IR spectra (Figure S4) confirmed the presence of aromatic rings (breathing vibrations at 1460–1600 cm^–1^) and alkyl chains (C–H stretches at 2755–3026 cm^–1^).^30^ The strong peak at ∼810 cm^–1^ originating from the out-of-plane bending vibration of C–H on the fluorene ring clearly indicates the fluorene ring. The asymmetric stretching vibration of the C–S–C bonds of thiophene at ∼793 cm^–1^ was evident in BT–Fl and BT–Fl–Py. Critically, BT–Fl–Py displayed enhanced peaks at 1430 and 1607 cm^–1^, attributable to the ring skeleton vibrations of pyridine, confirming the successful incorporation of the pyridine acceptor. X-ray photoelectron spectroscopy (XPS) further validated the elemental composition and bonding environments of all polymers (Figures S5–S7).

**Figure 1:**
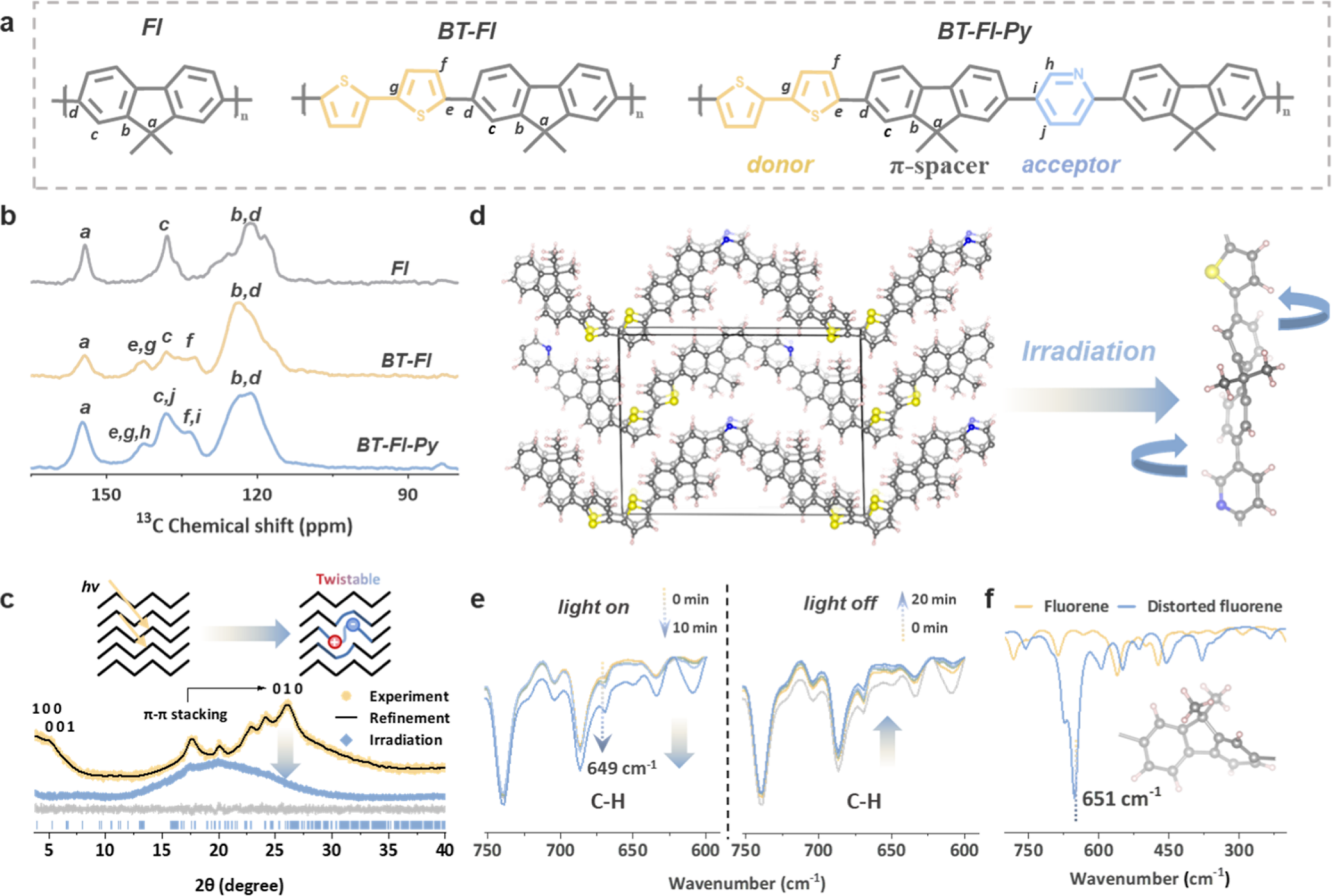
Structure of the BT–Fl–Py polymer. (a) Chemical structures of the conjugated polymer photocatalysts. (b) Solid-state ^13^C NMR spectra of Fl, BT–Fl, and BT–Fl–Py polymers. (c) Pawley refinement with the residual difference plot and the XRD pattern of the BT–Fl–Py polymer before and after irradiation. The schematic diagram shows the formation of light-triggered torsional distortion. (d) Refined crystal lattice structure of the BT–Fl–Py polymer. (e) FTIR spectra of the BT–Fl–Py polymer recorded in the dark and under light irradiation. (f) Calculated infrared vibration of the distorted fluorene.

To probe the structural change under light irradiation, we conducted in situ illumination studies. X-ray diffraction (XRD) patterns of BT–Fl–Py revealed a dramatic response to light (Figure 1c). The disappearance of the (100) and (001) peaks indicates a complete loss of both side-chain ordering and interchain periodicity, signaling a substantial photoinduced molecular rearrangement. Concurrently, the broadening and weakening of the (010) peak (π–π stacking) suggest that while short-range face-to-face interactions are partially retained, they are under significant lattice strain due to localized polaronic distortions. Taken together, these observations support a light-triggered torsional distortion along the backbone that accompanies the charge redistribution between the bithiophene donor and the pyridine acceptor. As visualized by the computational model in Figure 1d, the fluorene π-spacer undergoes pronounced twisting along the molecular axis after photoexcitation, which reduces effective conjugation.

Further evidence of photoinduced structural modification was gleaned from FT-IR spectroscopy. Enhanced intensities in the wavelength regions of 700–750 cm^–1^ (C–S stretching), 1450–1562 cm^–1^ (aromatic C=C/C–C vibrations), and 1900–2050 cm^–1^ (characteristic of charge-separated species) collectively point to increased conjugation disruption, charge localization, and the formation of a distinct electronic state. Importantly, the gradual decline of these signals after stopping illumination indicates the reversibility of the torsion structure (Figures 1e, S9). The appearance of a new band at ∼649 cm^–1^ is assigned to the out-of-plane bending mode of distorted fluorene rings verified by theoretical calculation (Figure 1f), supporting the occurrence of light-triggered torsion along the π-bridge.

This conclusion is further supported by XPS measurements under light illumination (Figure S10). A pronounced shift of the N 1s XPS peak to a lower binding energy (−0.43 eV) indicates an increased electron density on the pyridinic nitrogen, whereas an upward shift of the S 2p XPS peak (+0.27 eV) reveals electron depletion from the thiophene donor. Collectively, these results indicate that the photoinduced twisting is electronically driven and coupled to donor-to-acceptor charge-transfer excitation in the D−π–A backbone. Such charge-transfer-coupled torsional relaxation is characteristic of a twisted intramolecular charge-transfer (TICT) configuration. We therefore employ time-resolved and steady-state spectroscopy to identify the long-lived TICT species (TICT_1_) and quantify its population buildup upon irradiation.

### Photogenerated Long-Lived TICT_1_ State as the Active Reducing Species

We next delineate the photophysical pathway that couples photoinduced torsion to the formation of a long-lived charge-separated state with a potent reducing ability. Steady-state UV–vis difference spectra track the buildup of the photoinduced species under continuous irradiation (Figure 2a). Upon irradiation, the ground-state absorption at ∼430 nm is bleached, concomitant with the emergence of a pronounced new band centered at ∼360 nm and a weak, broad feature spanning 650–750 nm. Well-defined isosbestic points persist throughout the irradiation sequence (Figures S11 and S12), indicating a clean interconversion rather than uncontrolled photodegradation. The spectral evolution is largely insensitive to solvent and interchain effects (Figures S13 and S14), supporting an intramolecular origin of the photoinduced state. The spectrum partially recovered upon storage in the dark (Figure S16), consistent with a metastable, yet reversible, photoinduced state. Notably, neither BT–Fl (Figure S17) nor Fl (Figure S18) shows detectable spectral evolution under identical conditions, underscoring the essential role of the pyridine acceptor in enabling this photophysical pathway.

**Figure 2:**
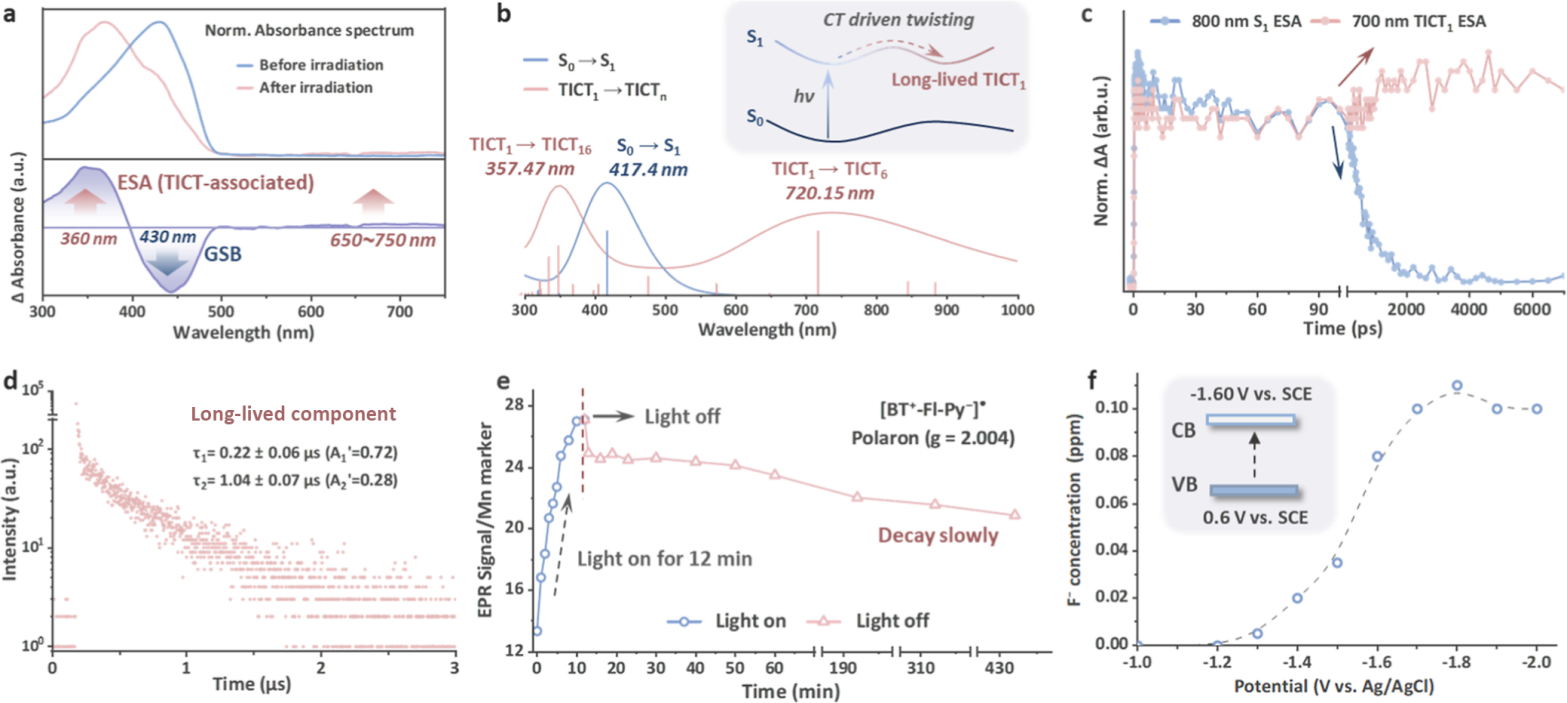
Formation and characterization of the long-lived TICT_1_ state. (a) Normalized steady-state absorption spectra of BT–Fl–Py before and after irradiation (upper part). Light-induced normalized absorption difference spectrum (lower part). GSB: ground-state bleaching; ESA: excited-state absorption. (b) TD-DFT simulated spectra of BT–Fl–Py. Blue: ground-state (S_0_) absorption, dominated by the lowest-energy S_0_ → S_1_ transition. Red: calculated light-induced absorption features associated with the optimized net-charged TICT_1_-related model, used to approximate the oxidized/hole-accumulated photoinduced species. Spectra were convoluted with Gaussian broadening (see Supporting Information for computational details). Inset: schematic of single-photon excitation, followed by torsional relaxation from S_0_ → S_1_ to the twisted charge-separated configuration (TICT_1_). (c) Delay kinetics of TICT_1_-associated and S_1_-associated ESA signals of BT–Fl–Py in THF (λ_ex_ = 420 nm). (d) Transient photoluminescence (TRPL) decay curve of the BT–Fl–Py polymer, focusing on the long-lived component (λ_ex_ = 405 nm). Measurement conditions: ambient air atmosphere, room temperature, time window = 0–3 μs. Fitting parameters (biexponential): τ_1_ = 0.22 ± 0.06 μs (normalized relative amplitude A_1_′ = 0.72), τ_2_ = 1.04 ± 0.07 μs (normalized relative amplitude A_2_′ = 0.28). A_1_′ and A_2_′ sum to 1, representing the relative contribution of each decay component. (e) Ratio of double-integral area of the in situ EPR signal of BT–Fl–Py powder to the Mn-marker signal in the dark and under light irradiation. (f) Dependence of F^–^ concentration on applied potential (vs Ag/AgCl) during PFOA degradation. Inset: energy band structure of BT–Fl–Py.

To further understand the factors that modulate the steady-state distribution of photoinduced species, we performed atmosphere-dependent steady-state absorption measurements. After 5 min irradiation, the signal at ∼360 nm is significantly stronger in air than in N_2_ (Figure S15). We attribute this enhancement to electron scavenging by O_2_, which extracts photogenerated electrons from the initially formed TICT_1_ state and thereby shifts the steady-state distribution toward a more persistent oxidized and hole-accumulated photoinduced species. Therefore, the stronger ∼360 nm absorption observed in air should be understood as reflecting an altered steady-state distribution of photoinduced species under the O_2_-rich conditions, rather than simply a higher population of the reactive TICT_1_ state itself.

We then assign the origin of the ∼360 and 650–750 nm bands to establish the nature of this photoinduced species. TD-DFT reproduces the main S_0_ → S_1_ absorption of the ground-state fragment at 417.4 nm (blue trace, Figure 2b). Starting from the experimentally established light-triggered π-bridge torsion, we optimized a twisted geometry and calculated the corresponding TICT_1_ state by TD-DFT. To approximate the experimentally observed photoinduced charged species, a net-charged model was employed in the calculation. The simulated light-induced absorption spectrum associated with TICT_1_ exhibits two prominent bands at 357.5 and 720.2 nm, arising predominantly from the TICT_1_ → TICT_16_ and TICT_1_ → TICT_5_ transitions, respectively (red trace, Figure 2b). The close correspondence between the calculated spectrum and the experimental ∼360 and 650–750 nm supports the assignment of these light-induced absorption features to the TICT_1_-related photoinduced state.

Femtosecond transient absorption (fs-TA, 420 nm excitation) further elucidates the TICT_1_ formation dynamics (Figure S19). Two ESA windows centered at ∼700 and ∼800 nm evolve in an anticorrelated manner: the ∼700 nm band decays sharply at ∼180 ps, while the ∼800 nm feature grows gradually (Figure 2c). This kinetic interplay is consistent with the population flow from the initially formed S_1_ to the torsion-driven TICT_1_ state. The ∼800 nm band persists beyond the measurement time scale, in stark contrast to the fluorene control (Figure S20). Consistently, transient photoluminescence (TRPL) shows accelerated depopulation of the emissive singlet manifold in BT–Fl–Py, with a shortened nanosecond component (τ_2_ = 2.6 ns) relative to BT–Fl (6.08 ns) and Fl (26.53 ns) (Figure S21), evidencing a fast nonradiative channel that competes effectively with radiative decay from S_1_. Moreover, BT–Fl–Py exhibits a distinct microsecond-lived component (τ = 1.04 μs; Figure 2d), consistent with relaxation into a long-lived torsion-stabilized CT state. This prolonged lifetime provides a sufficient temporal window for interfacial electron transfer from the TICT_1_-related state to PFOA before charge recombination rather than implying a required sequential photoexcitation process.

In situ EPR measurements independently corroborate the formation of a long-lived charge-bearing species with polaronic character. Under illumination, BT–Fl–Py displays a pronounced signal at *g* = 2.004 (Figures 2e, S22). The intensity increases with irradiation time and reaches a plateau after ∼12 min, indicating the establishment of a steady-state paramagnetic population under continuous light irradiation. After switching off light, the signal decays slowly and remains detectable even after 7 h, evidencing the persistence of a long-lived photoinduced charge-bearing species consistent with the TICT_1_-related state. In contrast, Fl and BT–Fl show negligible signals under identical conditions (Figure S23). Taken together with the reversible difference spectra, these results support direct light-driven access to a long-lived TICT_1_-related state without externally added sacrificial agents.

The BT–Fl–Py polymer exhibits a ground-state reduction potential (corresponding to the conduction band, *E*_CB_) of −1.6 V vs SCE (Figures 2f inset, S24–S28). To elucidate the thermodynamic feasibility of reductive defluorination of PFOA by our photocatalyst, we systematically examined the release of F^–^ from PFOA under a range of applied electrochemical potentials. A sharp increase in F^–^ concentration was observed at −1.4 ∼ −1.8 V vs Ag/AgCl (Figure 2f), marking the onset of reductive C–F bond cleavage for PFOA. Notably, a catalyst-coated working electrode was employed to closely mimic the practical photocatalytic setup, thereby accounting for the specific intermolecular interactions between PFOA and the BT–Fl–Py polymer. Collectively, these results indicate that the reducing capability of BT–Fl–Py, together with catalyst–PFOA interfacial interactions, is sufficient to initiate interfacial reductive defluorination under the single-photon framework. Critically, the photoinduced formation of a long-lived TICT state stabilizes the photogenerated reducing electrons by suppressing rapid charge recombination while retaining sufficient reductive capability for interfacial electron transfer. Together, these results support the initially generated long-lived TICT_1_ state as the active reducing species in the single-photon pathway, without requiring sequential excitation of higher-lying TICT_*n*_ states.

### Visible-Light-Driven Photocatalytic Defluorination of PFOA by BT–Fl–Py

Defluorination efficiency was quantitatively assessed under standardized conditions: an initial PFOA concentration of 0.05 ppm in an additive-free aqueous solution under an air environment (dissolved O_2_ ≈ 8 mg L^–1^). Note that all photophysical characterization of BT–Fl–Py was conducted in THF, which was used to obtain homogeneous solution-phase photophysical data, while the photocatalytic defluorination of PFOA was performed in water to reflect the relevant aqueous reaction conditions. This difference is primarily due to solubility constraints, as BT–Fl–Py is poorly soluble in water and forms a slurry, making direct photophysical characterization in an aqueous environment impractical. A 6 h dark adsorption period ensured system equilibrium prior to light illumination, with the fluoride release monitored in real time by ion chromatography and a fluoride ion-selective electrode. BT–Fl–Py demonstrated exceptional adsorption kinetics, achieving complete PFOA removal from solution within 5 min across a concentration range of 0.05–0.5 ppm (Figure S29). Crucially, no fluoride ions were detected during this adsorption phase. Upon irradiation with visible light (λ ≥ 420 nm), a gradual accumulation of fluoride ions was observed. After 30 h, the fluoride concentration reached the theoretical maximum yield from the adsorbed PFOA (quantified as 0.0345 ± 0.005 ppm of F^–^; Figure 3b), confirming near-quantitative photocatalytic defluorination. The reliability of the PFOA and F^–^ mass balances is limited to the detection limits achievable using liquid chromatography and fluoride ion-selective electrodes. Control experiments confirmed the necessity of the photocatalyst, as negligible defluorination occurred via direct photolysis under identical conditions. Full-spectrum irradiation (360–780 nm) yielded a modest enhancement in the defluorination rate compared to visible light alone (30 h^–1^ vs 24 h^–1^). A relative photon absorption analysis further indicates that the polymer absorbs approximately 24.6% more photons under full-spectrum irradiation than under visible-light irradiation (see Supporting Information for details). The enhanced defluorination efficiency is therefore largely attributed to increased photon absorption. This high activity is supported by carrier lifetime and photoelectrochemical measurements (Figures S30–S41),^31,32^ which demonstrate that BT–Fl–Py provides an excellent platform for photocatalytic reactions.

**Figure 3:**
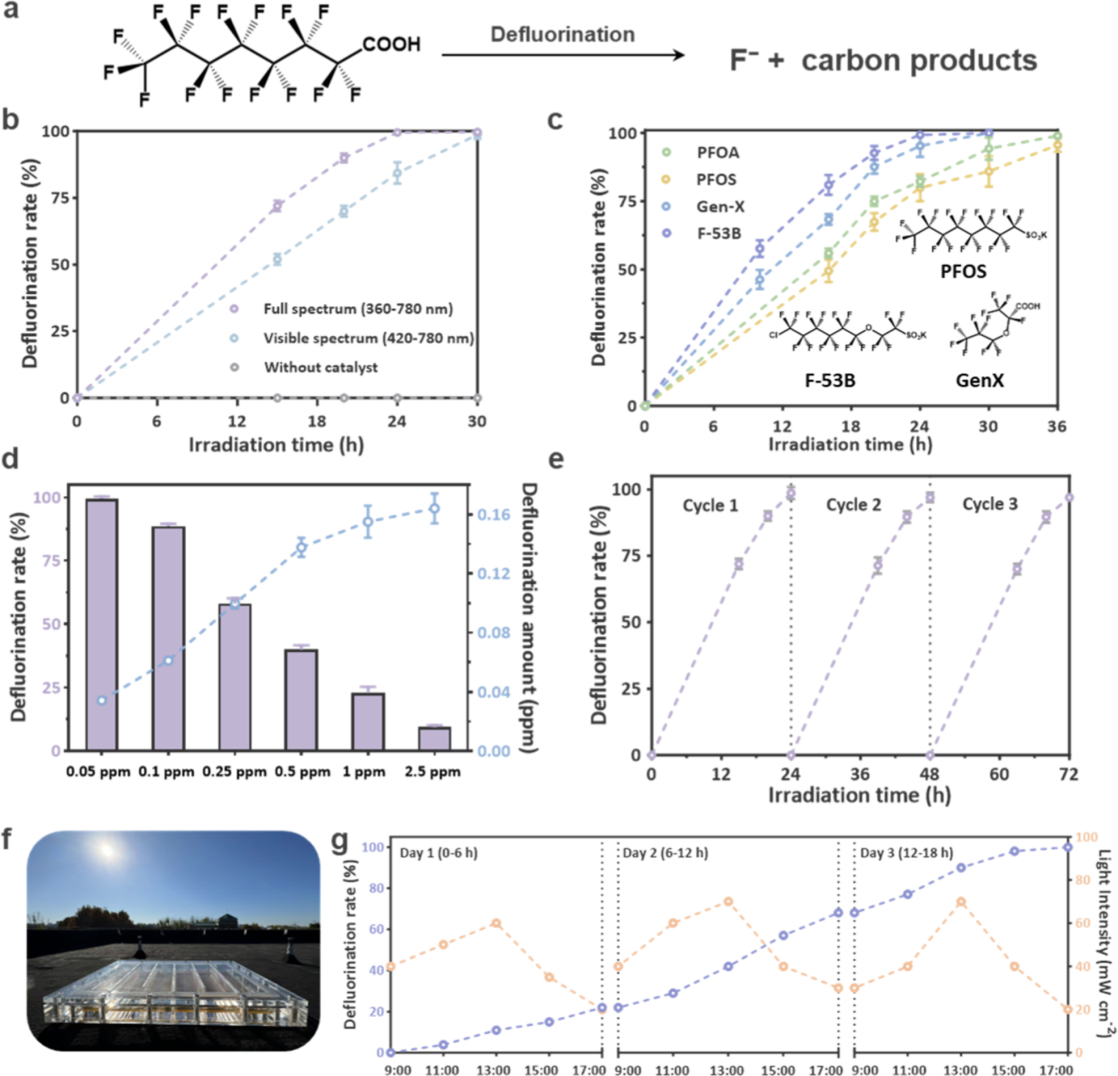
Photocatalytic reductive defluorination performance for PFAS. (a) Reaction mode of PFOA in the photocatalytic defluorination process. (b) Comparative wavelength-dependent activity: full-spectrum vs visible-light irradiation (0.05 ppm PFOA). (c) Substrate scope analysis of polyfluoroalkyl substances ([PFAS] = 0.1 ppm, catalyst loading = 0.2 g L^–1^) (d) Concentration-dependent defluorination efficiency under visible-light irradiation (PFOA: 0.05–2.5 ppm). (e) Long-term operational stability over 3 consecutive cycles (72 h total duration). (f) Custom-designed outdoor photoreactor and experimental setup for photocatalytic PFOA defluorination (reactor scale: 36 × 30 × 5 cm^3^). (g) Defluorination performance of BT–Fl–Py over 3 days of natural sunlight irradiation (6 h/day).

The substrate scope was extended to include four structurally diverse PFAS and their substitutes (Figure 3c). PFOA exhibited a marginally higher defluorination rate than PFOS. This divergence is rationalized by the relative lability of the headgroup bonds targeted by photogenerated holes for the initial activation step; the C–C bond in carboxylates is more readily cleaved than the stronger C–S bond in sulfonates. The defluorination efficiency of GenX and F-53B are higher than those of PFAS because the heteroatoms in the main chain weaken the shielding effect of the perfluoroalkyl chain, making C–F bonds easier to be attacked. Notably, the performance of BT–Fl–Py vastly surpasses that of classical thermal methods (e.g., alkaline treatment in DMSO, which achieved <1% defluorination for PFOS after 150 h^21^). Our photocatalytic system operates via a synergistic mechanism involving hydrophobic adsorption and direct hole-mediated headgroup cleavage, offering a markedly superior, milder, and more environmentally compatible alternative for degrading both PFCAs and PFSAs at trace concentrations.

The influence of initial PFOA concentration (0.05–2.5 ppm) on defluorination capacity was investigated (Figures 3d, S42). The total amount of released fluoride increased with the initial PFOA concentration, reaching an optimum capacity of 8.625 mg g^–1^ cat. at 2.5 ppm PFOA. The observed rate of defluorination, however, exhibited an inverse correlation with the initial PFOA concentration. This suggests a surface-mediated process where competitive inhibition by released fluoride ions may impede adsorption via electrostatic effect.

Besides, the BT–Fl–Py photocatalyst exhibited outstanding recyclability and stability (Figure 3e). Over three consecutive cycles, BT–Fl–Py maintained a defluorination efficiency exceeding 98% for 0.05 ppm of PFOA. Postreaction characterizations confirmed the preservation of the polymer’s morphological integrity (Figures S43 and S44), underscoring its robustness. To bridge the gap between the laboratory proof-of-concept and practical application, a pilot-scale evaluation was conducted using a custom solar reactor designed for real-world conditions (Figure 3f). As shown in Figure 3g, this system achieved complete (100%) PFOA defluorination in 3 days using only natural sunlight (6 h day^–1^ at ≤ 80 mW cm^–2^). This demonstration of solar-driven outdoor application, combined with the catalyst’s operational simplicity, absence of chemical additives, ultralow energy requirements, and metal-free composition, establishes BT–Fl–Py as a highly promising and scalable photocatalyst for sustainable PFAS remediation.

### Reaction Mechanism

Efficient electron transfer, a prerequisite for photocatalytic defluorination, is contingent upon a strong interfacial interaction between the catalyst and the substrate. A critical challenge in the aqueous system is the competition among water, oxygen, and the target pollutant (PFOA) for photogenerated electrons. The polymer skeleton of BT–Fl–Py, constructed from nonpolar polycyclic aromatic fluorene and rigid planar bithiophene units, creates a hydrophobic framework that impedes the penetration of water and oxygen molecules. This is further enhanced by the methyl side chains of the fluorene units, which provide additional steric hindrance. Water contact angle measurements give contact angles of 91.7°, 98.2°, and 102.1° for BT–Fl–Py, BT–Fl, and Fl, respectively (Figures 4a, S45). The moderate hydrophobicity of BT–Fl–Py is ideal, which can facilitate preferential adsorption of the hydrophobic perfluoroalkyl chain while maintaining sufficient contact with the aqueous medium and dispersion of the catalyst in water. Furthermore, the O_2_ temperature-programmed desorption (O_2_-TPD) curve showed almost no physical oxygen adsorption over BT–Fl–Py (Figures 4b, S46), using commercial graphene and UiO-66 as refs,^30,33^ indicating the material’s weak affinity for O_2_ toward competing reduction reactions. Note that photophysical characterizations were conducted in THF, where BT–Fl–Py is molecularly dissolved and, therefore, readily quenched by dissolved O_2_. In water, BT–Fl–Py is poorly soluble and forms aggregated solids in a heterogeneous suspension, which limits O_2_ accessibility and transport. As O_2_ competes with PFOA for photogenerated electrons, its influence under photocatalytic conditions is therefore expected to be substantially reduced compared to that observed in homogeneous THF photophysical measurements.

**Figure 4:**
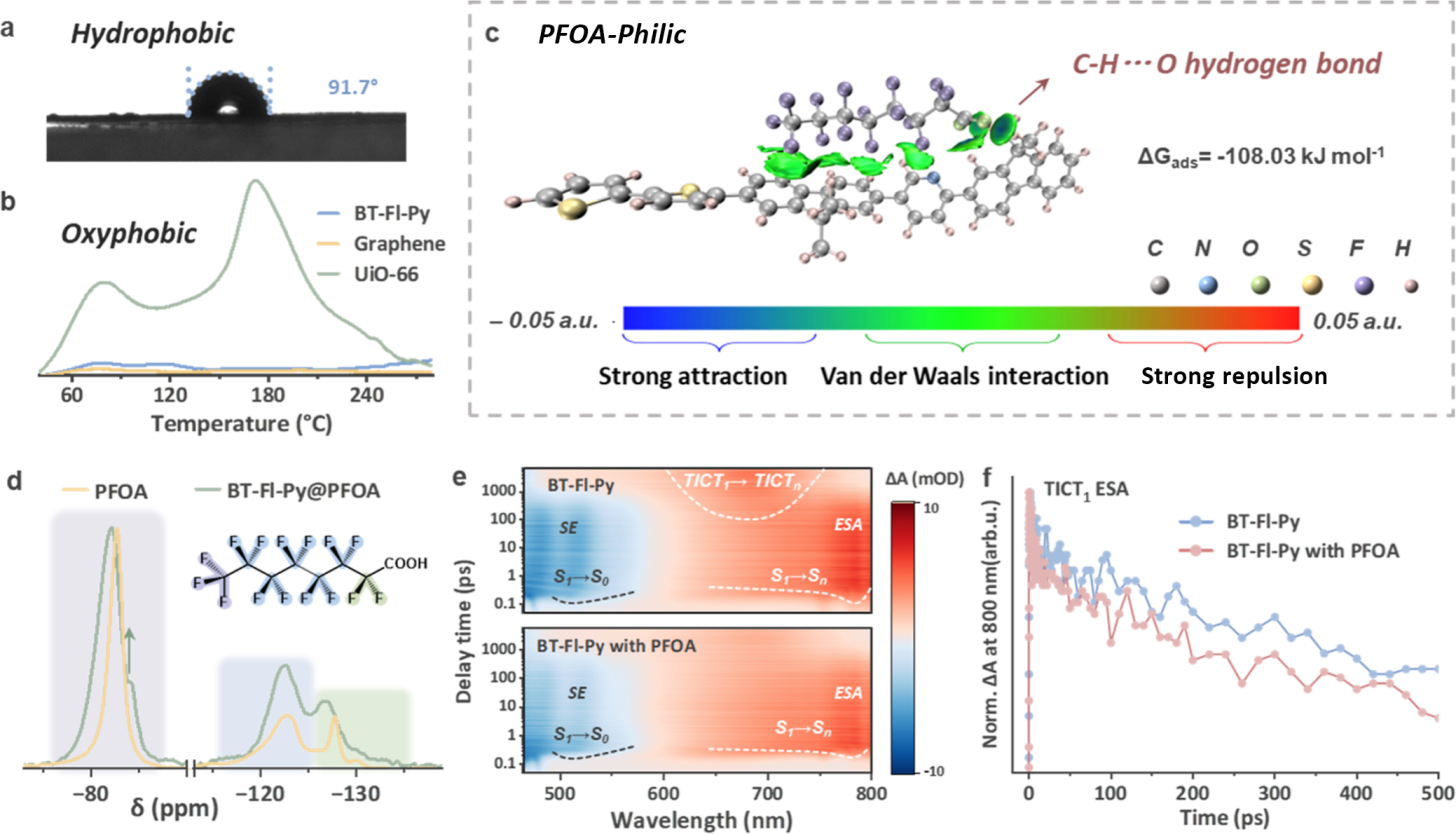
Preferential adsorption and activation of PFOA. (a) Water contact angle of BT–Fl–Py. (b) O_2_-TPD curves of BT–Fl–Py. (c) Independent gradient model based on the Hirshfeld partition (IGMH) analysis of the intermolecular interactions between PFOA^–^ and BT–Fl–Py. (d) Solid-state ^19^F NMR spectra showing adsorption-induced vibrational shifts. (e) Femtosecond transient absorption spectra of BT–Fl–Py and BT–Fl–Py with PFOA in THF. (λ_ex_ = 420 nm). (f) Extraction of the kinetics curves of the TICT_1_ ESA signal.

In a neutral aqueous solution, the carboxyl group of PFOA is in a completely deprotonated state (p*K*_a_ ≈ 0.5), mainly existing in the form of perfluorooctanoate (PFOA^–^). The independent gradient model based on Hirshfeld partition (IGMH) analysis^34,35^ was used to visualize the interaction between BT–Fl–Py and PFOA^–^. As shown in Figure 4c, weak hydrogen bonds (blue isosurface) between the C–H bonds of the BT–Fl–Py skeleton and the carboxyl oxygen atoms (C–O···H) are present in the adsorption system, while van der Waals interactions (green isosurface) can be seen between the fluorinated chain of PFOA ion and C–H groups on the catalyst. Therefore, hydrophobic and electrostatic interactions jointly facilitate the adsorption of PFOA by BT–Fl–Py in an aqueous environment. Also, based on density functional theory (DFT) calculations, the adsorption energy (Δ*G*_ads_) between PFOA^–^ and BT–Fl–Py was determined to be −108.03 kJ mol^–1^, indicating a strong adsorption tendency.

This intimate adsorption interface directly activates the C–F bonds of PFOA, priming them for reduction as probed by FT-IR spectroscopy (Figure S47) and ^19^F NMR (Figure 4d). Upon adsorption, the characteristic C–F stretching vibration of PFOA undergoes a pronounced blue shift from 1198 cm^–1^ to 1211 cm^–1^, indicating a change in the chemical environment of the C–F bonds. In addition, the normalized solid-state ^19^F NMR spectral peaks all showed significant broadening—a typical feature for the adsorbed state. The appearance of a new, shielded resonance at ∼84 ppm indicates that the terminal −CF_3_ group experiences a strong ring current effect generated by the aromatic system on the catalyst’s surface.^36^

The electron-transfer interaction between BT–Fl–Py and PFOA was further interrogated by femtosecond transient absorption spectroscopy (fs-TAS; Figures S19 and S48). As shown in Figure 4e, introducing PFOA almost completely suppressed the TICT_1_-associated ESA. Consistently, the kinetic trace at 800 nm—assigned to the TICT_1_ ESA—decayed substantially faster upon the addition of PFOA (Figure 4f). We attribute this accelerated decay to electron transfer from the photoexcited TICT_1_ state to PFOA: PFOA acts as an efficient electron acceptor, providing a direct depletion channel for the TICT_1_ population by accepting electrons from the TICT_1_ state. The accelerated decay of the TICT_1_-associated ESA signal is therefore strongly consistent with the electron transfer from TICT_1_ to PFOA, in line with the single-photon mechanism in which TICT_1_ directly mediates reductive C–F bond cleavage via interfacial electron transfer. In addition, we speculate that the photogenerated holes, which are concomitantly formed with electrons upon photoexcitation, may also participate in the oxidative decarboxylation of PFOA under continuous illumination. Such a process could cooperate with TICT_1_-mediated reductive C–F bond cleavage, thereby facilitating the overall degradation of PFOA.

To identify the main reactive species involved in the degradation of PFOA, AgNO_3_ and KIO_3_ were used as electron quenchers and EDTA-2Na as a hole sacrificial agent. AgNO_3_ and KIO_3_ directly deprived BT–Fl–Py of its defluorination ability, while EDTA-2Na reduced the defluorination performance (Figure 5a). These indicate that the photogenerated electron (e^–^) is the dominant active species that directly drives the defluorination process, while the photogenerated hole assists in some processes, such as the oxidative decarboxylation reaction.

**Figure 5:**
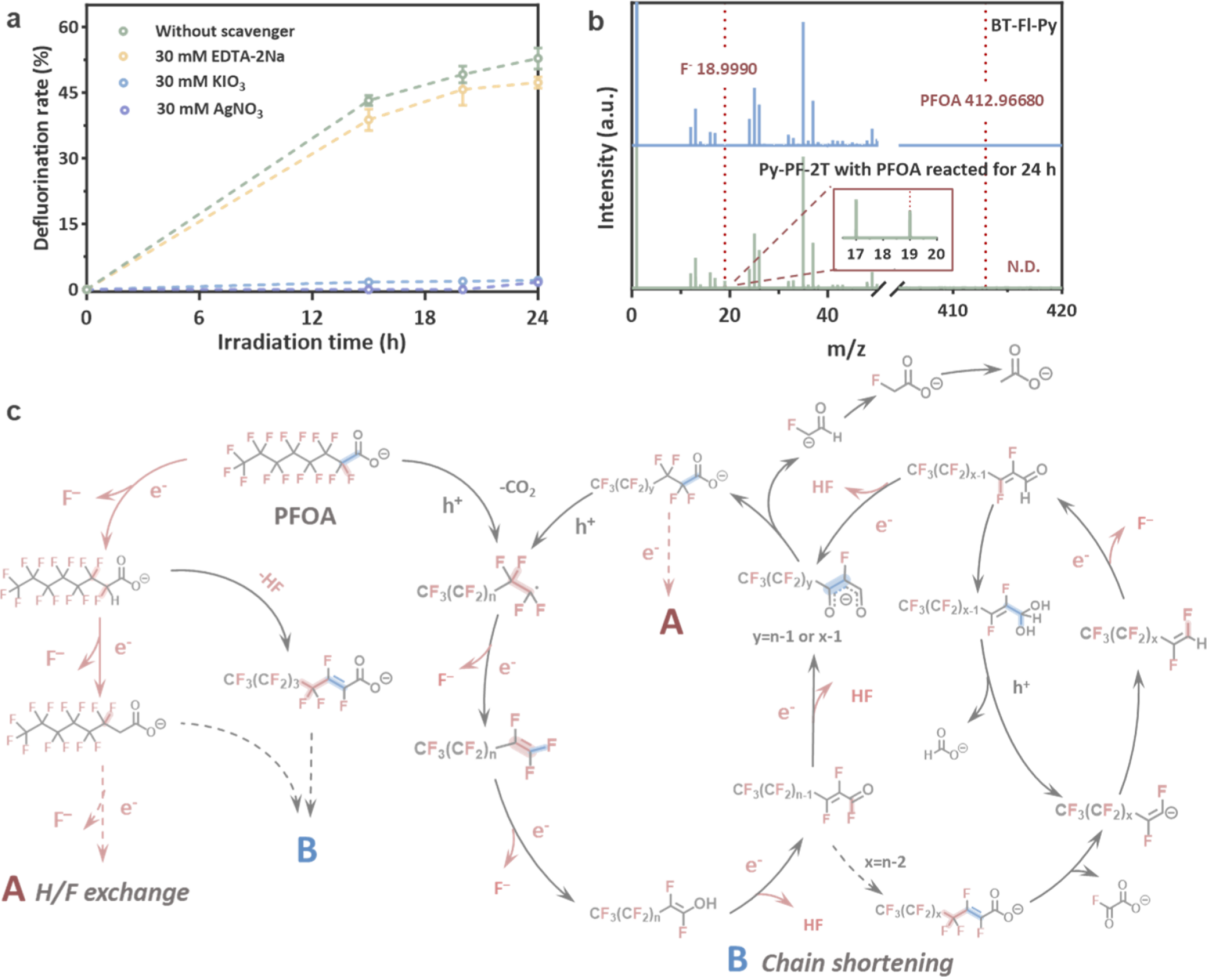
Possible defluorination and degradation mechanism for PFOA. (a) Defluorination kinetics of 0.25 ppm PFOA with different scavengers using BT–Fl–Py under full-spectrum irradiation during 24 h. (b) Negative-ion TOF-SIMS spectra of BT–Fl–Py before and after 24 h of photocatalytic reaction of 0.05 ppm PFOA under visible-light irradiation. (c) Proposed possible PFOA degradation pathway.

Liquid chromatography–mass spectrometry (LC–MS) analysis of the degradation intermediates identified shorter-chain perfluorocarboxylic acids (PFCAs, e.g., C_3_F_7_COO^–^, C_6_F_11_COO^–^), perfluoroalkyl anions (e.g., C_7_F_15_^–^), and hydrogen-substituted fluorocarboxylic acids (e.g., C_7_F_12_H_3_COO^–^) (Figures S49 and S50, Table S2). Note that the ion corresponding to C_7_F_15_^–^ (*m*/*z* 369) is consistent with the well-established fragmentation behavior of C_7_F_15_COO^–^ (*M*_W_ = 413), according to U.S. EPA Method 1633A and relevant reports.^37^ However, time-course experiments (Figure S51) show that its formation correlates with the photocatalytic degradation process, suggesting that decarboxylation occurs as a step generating a transient perfluoroalkyl anion intermediate, which rapidly protonates in the aqueous phase.^21^ Based on this, a degradation pathway is proposed, which is initiated by direct electron attack, leading to H/F exchange concurrent with hole-driven decarboxylation. The synergistic effect of this pathway—e^–^-mediated defluorination and h^+^-mediated chain-shortening—continuously propagates the degradation kinetic process (Figure 5c). Furthermore, fluoride mass balance indicates (near)­quantitative defluorination of PFOA. LC–MS and carbon analysis show that, within the examined reaction time window, the carbon is converted partly to inorganic carbon (IC, e.g., CO_2_/HCO_3_^–^/CO_3_^2–^) and partly to small nonfluorinated carboxylates (e.g., lactic acid and other short-chain carboxylic acids) (Figures S52 and S53). The accumulation of these small carboxylates reflects their slower subsequent oxidation/mineralization under heterogeneous aqueous photocatalytic conditions rather than indicating that they are intrinsically oxidation-resistant.

Time-of-flight secondary ion mass spectrometry (TOF-SIMS) analysis of the postreaction BT–Fl–Py catalyst detected adsorbed fluoride ions (F^–^) with no residual PFOA or other fluorinated organics, confirming effective substrate degradation (Figure 5b). The presence of adsorbed F^–^ accounts for the minor mass balance error observed in the defluorination assay.

## Conclusion

In summary, we developed a BT–Fl–Py polymer as a visible-light photocatalyst for the complete defluorination of environmental PFAS. Upon single-photon excitation, the polymer’s fluorene π-bridge undergoes torsional relaxation to form a long-lived TICT_1_ state, which stabilizes photogenerated electrons to enhance charge utilization. Concomitant hydrophobic backbone of the polymer enriches PFOA at the interface, thereby activating the C–F bond and facilitating defluorination without externally added sacrificial agents. Our strategy drives complete defluorination within 18 h under natural sunlight, demonstrating broad-spectrum defluorination efficacy across different PFAS. This study has successfully realized the nontoxic and harmless treatment of PFAS by using a mild and sustainable photocatalytic technique without externally added sacrificial agents.

## Supplementary Material



## Notes

Experiment section; characterization of photocatalysts; excitation analysis of photocatalysts; adsorption model for PCs and PFOA; and product analysis for the photocatalytic defluorination process of PFOA (PDF)

## #

Department of Environmental Science and Engineering, University of Science and Technology of China, Hefei, China
