# Supplementary material for: Photocatalytic Defluorination of Perfluorooctanoic Acid by Twisted Linear Polymer Radicals
Source: J Am Chem Soc. 2026 May 13;148(20):20364–73. doi: 10.1021/jacs.5c17497 (PMC13220261; doi:10.1021/jacs.5c17497)
Supplement: Supplementary file 1 [file ja5c17497_si_001.pdf]

Supporting Information

Photocatalytic defluorination of perfluorooctanoic acid by twisted linear polymer radicals

Jiaxi Hu[a], Yan Guo[b,*], Qixin Zhou[a,c], Ling Zhang[a], Haoying Wang[a], Junshan Li[e], Bin Liu[c,d,*] and Yongfa Zhu[a,*]

[a]Department of Chemistry, Tsinghua University, Beijing 100084, China

[b]Department of Civil Engineering, The University of Hong Kong, Hong Kong SAR 999077, China

[c]Department of Materials Science and Engineering, City University of Hong Kong, Hong Kong SAR 999077, China

[d]Department of Chemistry, Hong Kong Institute for Clean Energy Center (HKICE), Center of Super-Diamond and Advanced Films (COSDAF), City University of Hong Kong, Hong Kong SAR 999077, China

[e]Institute for Advanced Study, Chengdu University, Chengdu 610106, P. R. China

Supplementary for experiment method

Reagents and materials

Reagents were purchased from commercial sources of Sigma Aldrich, Alfa Aesar and used without further purification unless otherwise stated. 5,5’-Dibromo-2,2’-bithiophene, 2,5-dibromopydine, 2-(9,9’-dimethyl-7-(4,4,5,5-tetramethyl-1,3,2,-diaxaborolane) were purchased from Mackin Co., LTD. Perfluorooctanoic acid (PFOA), perfluorooctane sulfonic acid (PFOS), 6:2 chlorinated polyfluorinated ether sulfonate (F-53B), and 2-(heptafluoropropoxy)-2,3,3,3-tetrafluoropropanoate (GenX) were obtained from J & K Scientific Co., LTD.

Synthesis of photocatalysts (PCs)

The fluorene (Fl), bithiophene-fluorene (BT-Fl) and bithiophene-fluorene-pyridine (BT-Fl-Py) polymers were prepared by SUZUKI reaction, referencing to previous reports with some modifications[1-2].

Synthesis of Fl polymer

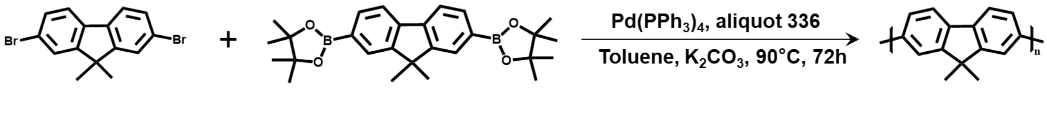

Scheme 1. Synthetic route of Fl polymer.

Under N_2 protection, 2,7-dibromo-9,9-dimethylfluorene (0.2 mmol, 70.4 mg), 9,9-dimethylfluorene-2,7-diboronic acid bis (pinacol) ester (0.2 mmol, 89.2 mg), and $Pd(PPh_3)_4$ (0.008 mmol, 10 mg) were added into a mixed solution containing 6 mL degassed toluene, 2 mL potassium carbonate aqueous solution (2 M) and one drop of Aliquat 336. The reaction system was heated to 90°C and stirred in the dark for 72 h. After cooling to room temperature, the resulting polymer was resolved in dichloromethane. The solution was washed thrice with saturated brine, dried with anhydrous Na_2SO_4, and then concentrated to an appropriate volume. The polymer was obtained by pouring the concentrated solution into methanol. The final purification was carried out by Soxhlet extraction with acetone for 24 h. After drying in vacuum at 60°C for 12 h, the polymer Fl was obtained as insoluble powder.

Synthesis of bithiophene-fluorene (BT-Fl) polymer

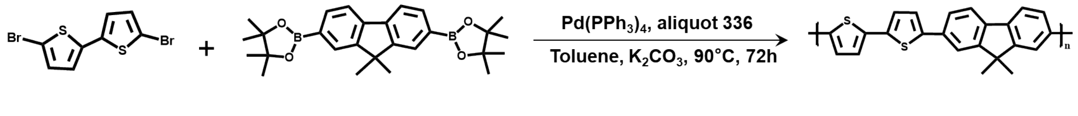

Scheme S2. Synthetic route of photocatalyst of BT-Fl.

Under N_2 protection, 5,5'-dibromo-2,2'-bithiophene (0.2 mmol, 64.8 mg), 9,9-dimethylfluorene-2,7-diboronic acid bis (pinacol) ester (0.2 mmol, 89.2 mg), and $Pd(PPh_3)_4$ (0.008 mmol, 10 mg) were added into a mixed solution containing 6 mL degassed toluene, 2 mL potassium carbonate aqueous solution (2 M) and one drop of Aliquat 336. The reaction system was heated to 90°C and stirred in the dark for 72 h.

After cooling to room temperature, the resulting polymer was resolved in dichloromethane. The solution was washed thrice with saturated brine, dried with anhydrous Na_2SO_4, and then concentrated to an appropriate volume. The polymer was obtained by pouring the concentrated solution into methanol. The final purification was carried out by Soxhlet extraction with acetone for 24 h. After drying in vacuum at 60°C for 12 h, the polymer BT-Fl was obtained as insoluble powder.

Synthesis of bithiophene-fluorene-pyridine (BT-Fl-Py) polymer

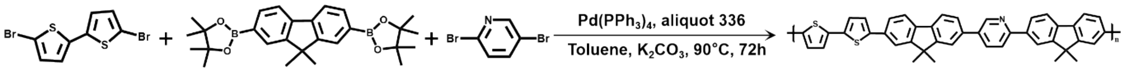

Scheme S3. Synthetic route of photocatalyst of BT-Fl-Py.

Under N_2 protection, 5,5'-dibromo-2,2'-bithiophene (0.1 mmol, 32.4 mg), 9,9-dimethylfluorene-2,7-diboronic acid bis (pinacol) ester (0.2 mmol, 89.2 mg), 2,5-dibromopyridine (0.2 mmol, 47.4 mg), and $Pd(PPh_3)_4$ (0.008 mmol, 10 mg) were added into a mixed solution containing 6 mL degassed toluene, 2 mL potassium carbonate aqueous solution (2 M) and one drop of Aliquat 336. The reaction system was heated to 90°C and stirred in the dark for 72 h. After cooling to room temperature, the resulting polymer was resolved in dichloromethane. The solution was washed thrice with saturated brine, dried with anhydrous Na_2SO_4, and then concentrated to an appropriate volume. The polymer was obtained by pouring the concentrated solution into methanol. The final purification was carried out by Soxhlet extraction with acetone for 24 h. After drying in vacuum at 60°C for 12 h, the polymer BT-Fl-Py was obtained as insoluble powder.

Characterizations

Fourier-transform infrared (FTIR) spectra were obtained on a Bruker V70 spectrometer. Light-induced IR was measured using a 300 W Xenon lamp (Microsolar300, Beijing Perfectlight) with full spectrum. The phase structure was analyzed by powder X-ray diffraction (PXRD) (Bruker Inc., Germany) with a Cu Kα radiation (λ = 1.54059 Å) on a Rigaku Smartlab X-ray diffractometer. Light-induced XRD patterns were recorded using a 300 W Xenon lamp source (Microsolar300, Beijing Perfectlight) with full spectrum. X-ray photoelectron spectroscopy (XPS) measurement was conducted on a Thermo ESCALAB 250 spectrometer with Al Kα X-ray radiation. *In situ* light-irradiated XPS spectra were recorded under a 440 nm laser (LSR-PS-II LASEVER INC.). The solid ^{13}C NMR spectra were recorded on a JEOL JNM-ECZ600R spectrometer at 298 K with the CPMAS method. The morphologies of the samples were investigated by field emission scanning electron microscopy (FESEM) on a Hitachi SU-8010 at an accelerating voltage of 10 kV, and transmission electron microscopy (TEM) on Hitachi HT 7700 at an accelerating voltage of 100 kV. Atomic force microscopy (AFM) experiments were performed on an Asylum Research Cypher VRS (Oxford instruments) atomic force microscope equipped with a Scan Asyst-HR fast scanning module and a Kelvin probe. Contact angle (CA) measurements were performed on the DSA 25 (KRUSS). The thermogravimetric analyses (TGA) were conducted on a Mettler Toledo TGA/DSC1 Star System analyzer under an air atmosphere at a heating rate of 5°C min^{-1} within the temperature range of 30~300°C. The ultraviolet-visible-near infrared diffuse reflectance spectroscopy (UV-Vis-NIR DRS) spectra were obtained on Agilent Cary 5000, using $BaSO_4$ as the reference. Photoluminescence (PL) spectra were obtained on the Edinburgh F900 spectrometer. PL lifetimes were measured using an FLS1000 fluorescence spectrometer (Edinburgh). Time-resolved photoluminescence (TRPL) spectra were measured using a time-correlated single photon counting (TCSPC) system (excitation wavelength: 405 nm). For the prompt fluorescence characterization, the measurement time window was set to 0~20 ns to capture fast decay processes; for the long-lived component analysis, the time

window was extended to 0~3 μs. All measurements were conducted under ambient air atmosphere at room temperature. Electron paramagnetic resonance (EPR) spectra were recorded on a JEOL FA-200 electron paramagnetic resonance spectrometer at room temperature. Light-induced EPR experiments were performed using a 300 W Xenon lamp source (Microsolar300, Beijing Perfectlight) with visible light ($\lambda > 420$ nm). TOF-SIMS was performed on a TOF.SIMS 5-100 (IONTOF GmbH). Perfluorooctanoic acid (PFOA) concentration was assessed via liquid chromatography-UV spectrometry (Agilent 6460). Chromatographic separation of sample was conducted using an XBridge C18 analytical column (150×3 mm, 3.5 μm), preceded by a Phenomenex Gemini C18 guard column (4 × 2 mm). High-resolution mass spectroscopy (HRMS) analysis was performed on a 6224 Time-of-Flight LC/MS (Agilent) at Colorado State University Central Instrument Facility. High-resolution mass spectrometry (HRMS) data were collected on a Bruker maxis UHR-TOF mass spectrometer in ESI positive mode.

Photoelectrochemical tests

The surface photovoltage (SPV) measurements were conducted on a home-built instrument. The monochromatic light resource was a 500 W Xenon lamp (CHF XQ500W) with a double-prism monochromator (Omni-λ 3005). The slit width was set at 3 mm. The photovoltage signal was amplified by a lock-in amplifier (SR830-DSP) with a light chopper (SR540). The resolution of the spectrum was 1 nm.

Electrochemical measurements were performed on an electrochemical system (CHI660E, Shanghai Chenhua Instrument Company). A standard three-electrode cell was employed with a working electrode, a platinum wire as the counter electrode, and an Ag/AgCl electrode as the reference electrode. 0.1 mol L^{-1} Na_2SO_4 aqueous solution was taken as the electrolyte and a 300 W Xe lamp with full spectrum was used as the light source. For the preparation of the working electrode, 2 mg photocatalyst was dispersed in 20 μL ethyl alcohol under ultrasonication to form a dispersion liquid. 20 μL of the resultant solution was then sprayed on the pretreated indium tin oxide with size of 1.0 × 1.0 cm^2 and dried at room temperature. The Mott-Schottky test was performed in the frequency range of 400 to 1000 Hz at a scan rate of 5 mV s^{-1}.

Controlled-potential experiments for PFOA defluorination were carried out at various constant potentials from −1.0 V to −2.0 V vs. Ag/AgCl for 10 min in 0.5 M $NaHCO_3$ aqueous solution containing 1 M PFOA. A three-electrode system was employed, consisting of a glassy carbon working electrode coated with the polymer photocatalyst, a platinum wire counter electrode, and an Ag/AgCl reference electrode. After electrolysis, the supernatant was collected, and the concentration of released F^- ions was determined using a fluoride ion-selective electrode.

PFOA adsorption experiment

A 25 mL aqueous solution containing 5 ppm PFOA was added to a Quartz sample tube, followed by adding 10 mg BT-Fl-Py, to form a slurry. During the stirring period, the mixture was filtered at intervals (0 min, 2 min, 5 min, 15 min, 30 min, 60 min, 90 min, and 120 min) through a 0.45 μm membrane filter for all samples, then the filtrates were analyzed by HPLC (Shimadzu LC-20AT) with a C18 reversed phase column (Venusil XBP-C18, 250 mm × 4.6 mm i.d., 5 μm) and a UV absorbance detector (K 2501) to determine the remaining PFOA content. The experiments were performed for three replicates. The experiments related to other comparing materials including Fl and BT-Fl were conducted based on the same procedure. The efficiency of pollutant removal by adsorption was determined using the following formula:

$$\text{PFOA removal rate} = \frac{C_0 - C_t}{C_0} \times 100\%$$

where C_0 (mg L^{-1}) and C_t (mg L^{-1}) are the initial and residual concentration of PFOA in the stock solution and filtrate, respectively.

Photocatalytic degradation of PFASs

The photodegradation reactions were performed in the XPA-7 photoreactor (Xujiang Power Plant, Nanjing, China). Typically, 5 mg photocatalyst powders were added into 25 mL PFOA aqueous solution in a 50 mL quartz tube with a magnetic stirrer. Before irradiation, the suspension solution was first ultrasonically dispersed for 10 min and then magnetically stirred for 30 min to reach the adsorption-desorption equilibrium. A 500 W Xenon lamp tube (360~780 nm, 70 mW cm^{-2}) was utilized as the light source to irradiate the reaction solution at room temperature, while the visible spectrum required an additional 420 nm cut-off filter. During reaction, 5 mL of solution was sampled at predetermined time intervals and filtrated with a 0.45 μm Millipore filter to remove the solid catalyst for the detection of fluoride ion (F^-) and the degradation products. All the experiments were repeated in triplicate to reduce system error.

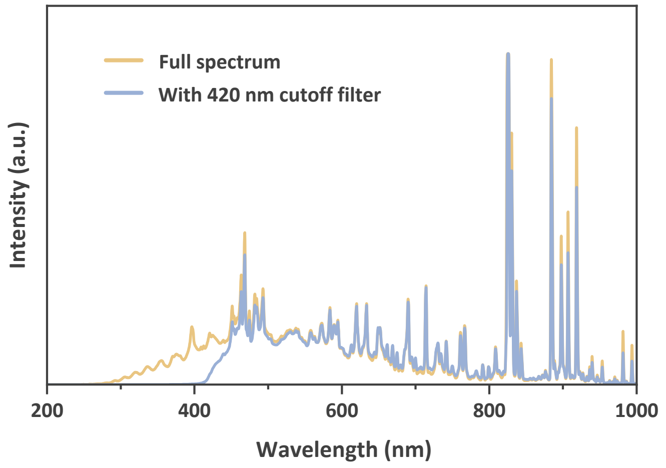

Figure S1. Spectrogram of the Xenon lamp used in this work.

Calculation of relative photon absorption

According to the fundamental principle of photon absorption, the number of photons absorbed by a sample (N_{abs}) can be calculated by integrating the product of the incident photon flux and the sample's absorption fraction over the wavelength range of interest:

$$N_{abs} = \int \frac{P(\lambda)\cdot \lambda}{hc} \cdot (1 - 10^{-A(\lambda)})\, d\lambda \qquad (1)$$

where *P(λ)* is the spectral power density of the light source, *A(λ)* is the absorbance of the sample, *h* is Planck's constant, and *c* is the speed of light.

Here, we used a relative calculation to compare photon absorption by the polymer under full-spectrum and visible-light irradiation conditions, rather than to determine an absolute photon count. In this case, the relative number of absorbed photons is proportional to:

$$N_{abs}^{relative} \propto \int I(\lambda) \cdot \lambda \cdot (1 - 10^{-A(\lambda)}) d\lambda \qquad (2)$$

where *I(λ)* is the relative intensity of the light source.

To perform the numerical integration, the continuous spectra were discretized at an interval of 10 nm over the range of 200~800 nm.

The integral in equation (2) was approximated by the following discrete summation:

$$N_{abs}^{relative} \propto \sum [I(\lambda_i) \cdot \lambda_i \cdot (1 - 10^{-A(\lambda_i)}) \cdot \Delta\lambda] \qquad (3)$$

where $I(\lambda_i)$ is the relative intensity at wavelength λ_i, and Δλ=10 nm is the sampling interval.

Using the convolution of the light source spectrum and the UV-vis DRS spectrum of the polymer, the relative absorbed photons under full-spectrum irradiation (N_{full}) and visible-light irradiation with a 420 nm cutoff filter (N_{vis}) were estimated. The calculation indicates that N_{full} is approximately 24.6% higher than N_{vis}, supporting the interpretation that the improved defluorination rate under full-spectrum irradiation mainly originates from increased photon absorption by the polymer.

Determination of fluoride ion concentration

The concentration of free F^- produced during photocatalytic degradation was determined using a fluoride ion selective electrode (ISE, PXSJ-216F, Leici, Shanghai) with Ag/AgCl as the reference electrode. Before each measurement, a linear relationship between the ISE response and the concentration of F^- standard solution was evident based on a standard curve that made with 5 concentrations of NaF standard solution. The standard sample or sample to be tested was mixed with TISAB as 1:1.

Preparation of buffer for total ionic strength adjustment (TISAB): 58.8 g sodium citrate and 85 g sodium nitrate were dissolved in 800 mL deionized water, and the pH was adjusted to 5.0~6.0 using hydrochloric acid, and diluted to 1000 mL with deionized water.

The defluorination rate was calculated as follows:

$$\mathrm{deF\%} = \frac{[\mathrm{F^-}]}{[\mathrm{PFAS}]_0 \times \mathrm{N_{C-F}}} \times 100\%$$

where $[F^-]$ is the molar concentration of F^- released in solution, [PFAS] is the initial molar concentration of the parent PFAS and $N_{C\text{-}F}$ is the number of C-F bonds in the parent PFAS molecule.

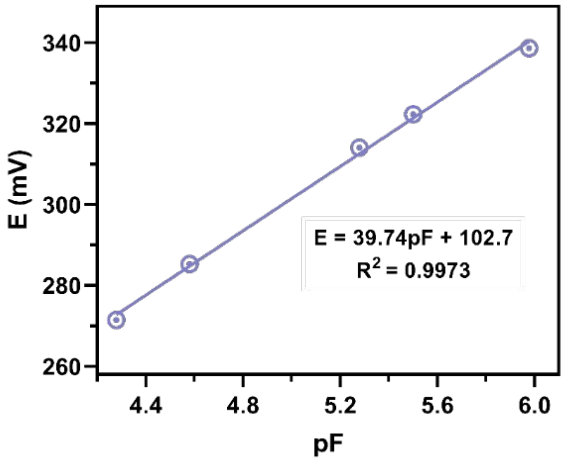

Figure S2. The standard curve of F^- measured by the fluoride ion selective electrode (pF represents –lg$[F^-]$).

Besides, the fluoride ion selective electrode was verified using ion chromatography (IC).

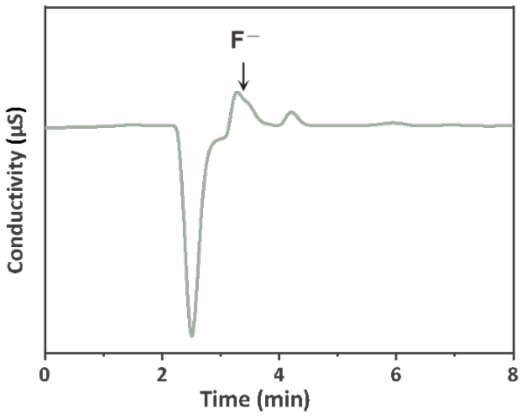

Figure S3. The IC signal for F^- in the degradation products of PFOA sample. (Sample: 1 ppm PFOA with BT-Fl-Py catalyst under full-spectrum irradiation for 24 h).

Transient absorption (TA) measurement

Transient absorption (TA) measurements were conducted using a pump-probe spectrometer setup. Initially, a Ti:sapphire amplifier generated a fundamental laser pulse with a wavelength of 800 nm. This fundamental pulse was then split into two branches by a beam splitter. One branch was directed towards an optical parametric amplifier to generate the pump pulse at 420 nm. The pump pulse, modulated at a frequency of 5000 Hz, underwent attenuation via neutral-density filter wheels. Simultaneously, the other branch of the fundamental pulse was focused onto a sapphire crystal to produce a white-light continuum spanning from 450 nm to 800 nm, utilized as the probe. Time delays between the pump and probe pulses were achieved using a motorized translation stage with a retro-reflecting mirror. The pump and probe beams were spatially overlapped on the sample surface, both being incident on the sample normally.

Theoretical calculations

All calculations were performed in Gaussian 16[3] using B3LYP/6-31G*. A truncated BT-Fl-Py segment was used as the model (net charge = +1). Geometry optimization of the $TICT_1$ state was performed at the (U)B3LYP/6-31G* level using an open-shell reference. Excited-state absorption was simulated by TD-DFT vertical excitations from the optimized TICT geometry to higher-lying excited states within the same manifold. Oscillator strengths were converted to a stick spectrum and broadened for comparison with the experimental transient absorption. Here, only the oscillator intensity of the charge-transfer excited component (corresponding to the charge-separated orbital characteristics of the S_1 state) is retained, while the intensity of the localized excitation is zeroed out.

The density functional theory (DFT) calculations for the model of PFOA and photocatalysts (PCs) were performed for geometry optimization, excited states and absorption spectra analysis. To analyze the weak interactions between the layers in the molecules, an independent gradient model based on the Hirshfeld partition (IGMH)[4-5] method in Multiwfn[6-7] was performed. The distribution of electrons and holes in the electron excitation process were also calculated and mapped using the Multiwfn program. The isosurface maps were rendered by the VMD program.[8]

To calculate the adsorption energy (ΔG_{ads}) between the photocatalysts and $PFOA^-$, the structure of photocatalysts, $PFOA^-$, and the adsorbed complex of PCs with PFOA were optimized with van der Waals dispersion correction. The adsorption energy was caculated based on the following equation:

$$\Delta G_{ads} = E_{PC\&PFOA^-} - (E_{PC} + E_{PFOA^-})$$

where $E_{PC\&PFOA}$, E_{PC}, and E_{PFOA} are the energies of PC after adsorption of $PFOA^-$, PC without adsorption, and $PFOA^-$, respectively.

Calculation of built-in electric field intensity

According to *Kanata-Kito* model[9-10], the built-in electric field of materials can be calculated as follows:

$$E = \sqrt{\frac{-2V_S\rho}{\varepsilon\varepsilon_0}}$$

where E represents the intensity of built-in electric field; V_s represents the surface potential detected via KPFM; ρ represents surface charge density obtained by the integral value of the current density; ε is the dielectric constant measured by the vector network analyzer; and ε_0 refers to the vacuum dielectric constant (8.854×10^{-23} $J\,K^{-1}$).

Characterization of PCs structures

The polymers were obtained as insoluble powder due to their rigid aromatic skeletons and crosslinked polymer chains. The successful synthesis of PCs was confirmed by Fourier transform infrared spectroscopy (FT-IR), solid-state ^{13}C nuclear magnetic resonance (NMR), X-ray diffraction (XRD), X-ray photoelectron spectroscopy (XPS) and SEM. FT-IR spectroscopy, solid-state ^{13}C NMR and XRD measurements were performed to identify the polymer structures, XPS was used to confirm the chemical compositions and SEM was used to examine the morphological information.

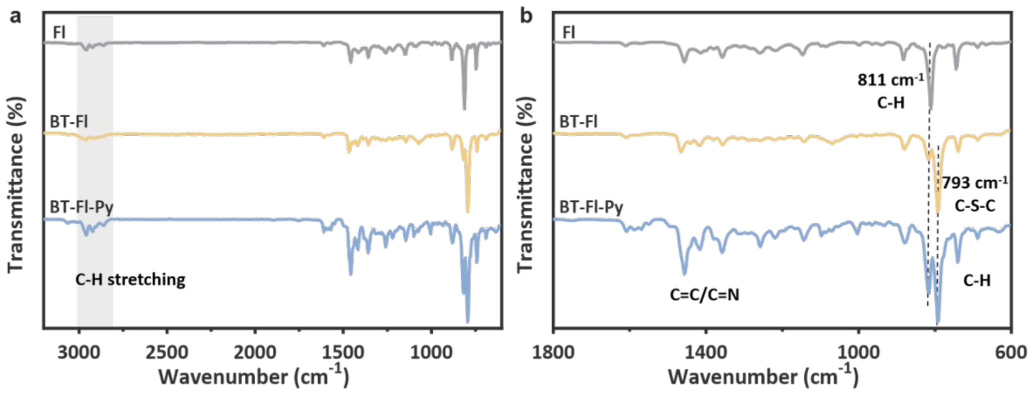

Figure S4. FT-IR spectra of the Fl, BT-Fl and BT-Fl-Py polymers.

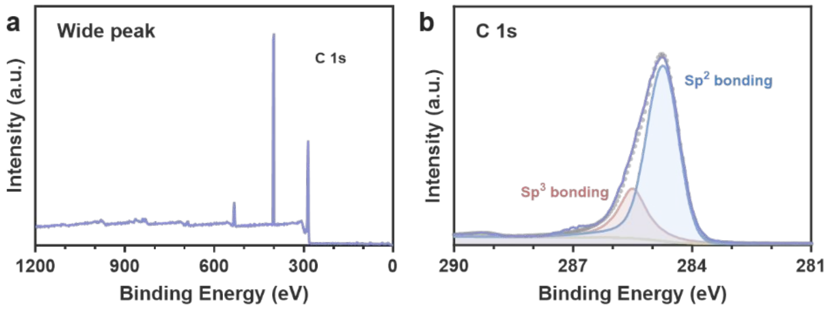

Figure S5. XPS analysis of Fl polymer. (a) XPS survey spectra and (b) high resolution C 1s XPS spectra.

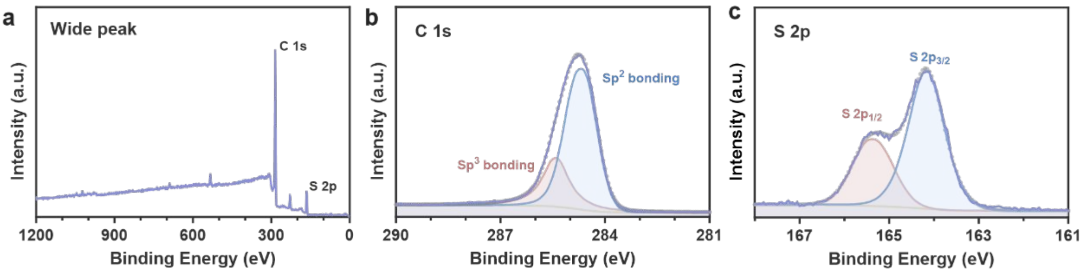

Figure S6. XPS analysis of BT-Fl polymer. (a) XPS survey spectra and (b, c) high resolution C 1s and S 2p XPS spectra.

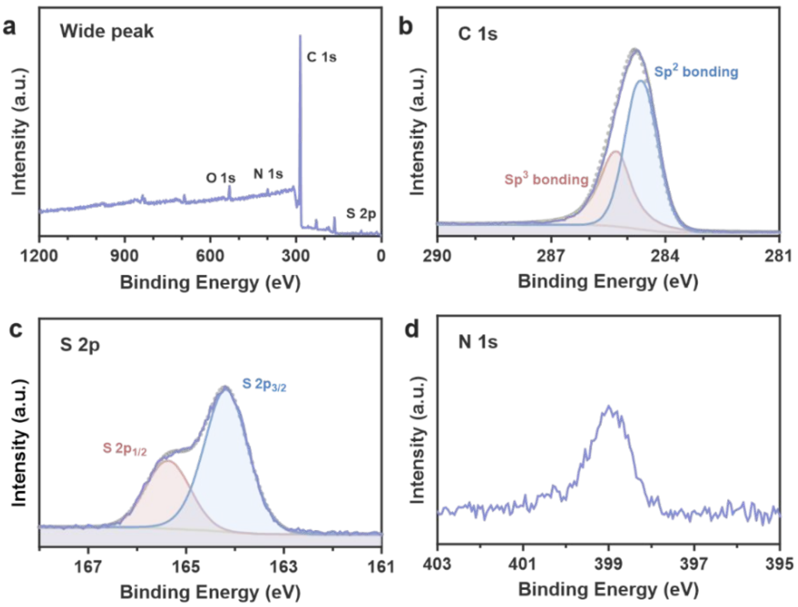

Figure S7. XPS analysis of BT-Fl-Py polymer. (a) XPS survey spectra and (b, c, d) high resolution C 1s, S 2p and N 1s XPS spectra.

XPS was used to confirm the chemical compositions of the Fl, BT-Fl and BT-Fl-Py photocatalysts as shown in **Figures S5-7**. Carbon element was detected in the three

polymer photocatalysts that could be fitted into two peaks (**Figures S5b-7b**). As widely reported, the main C 1s peak at 285 eV corresponded to the sp^2 aromatic carbon, and the weaker peak at 286 eV could be ascribed to sp^3 alkyl carbon due to the methyl group in Fl. Nitrogen element was only detected in BT-Fl-Py, and sulfur element was detected in BT-Fl-Py and BT-Fl, while neither nitrogen nor sulfur was detected in Fl. The N 1s peak positioned at 398.8 eV for BT-Fl-Py (**Figure S7d**), corresponding well to the hybridized aromatic N bonded to carbon atoms (C=N–C) from pyridine group, and the S 2p peak for BT-Fl and BT-Fl-Py (**Figure S6c and Figure S7c**) were in consistent with the reported S 2p XPS data.

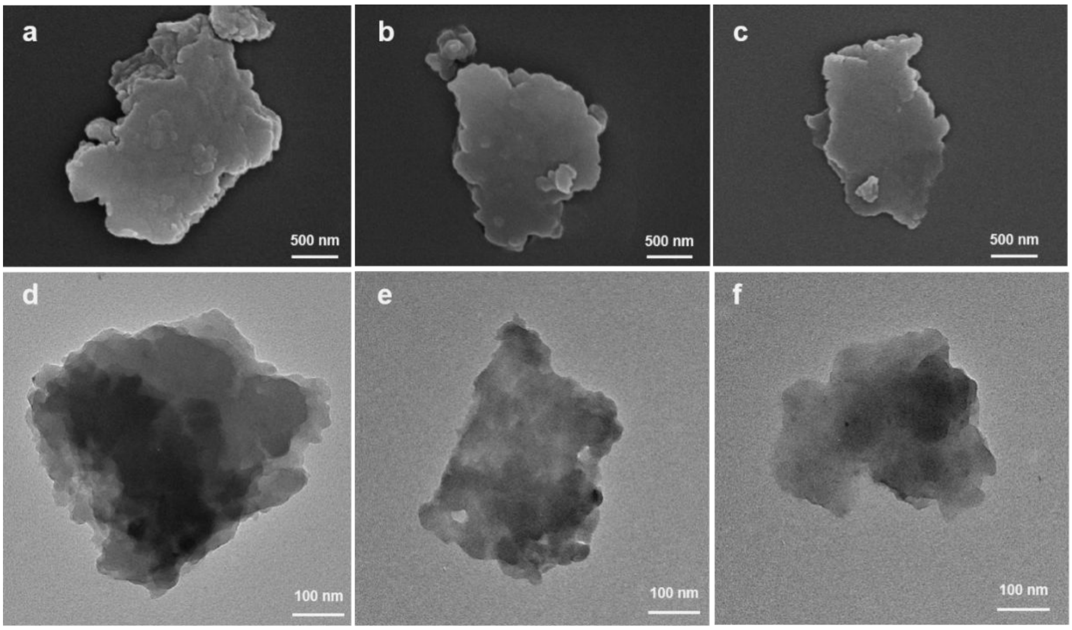

Figure S8. Morphology characterization of polymers. SEM images of (a) Fl, (b) BT-Fl and (c) BT-Fl-Py polymers; TEM images of (d) Fl, (e) BT-Fl and (f) BT-Fl-Py polymers.

Photo-induced polymer structure twisting

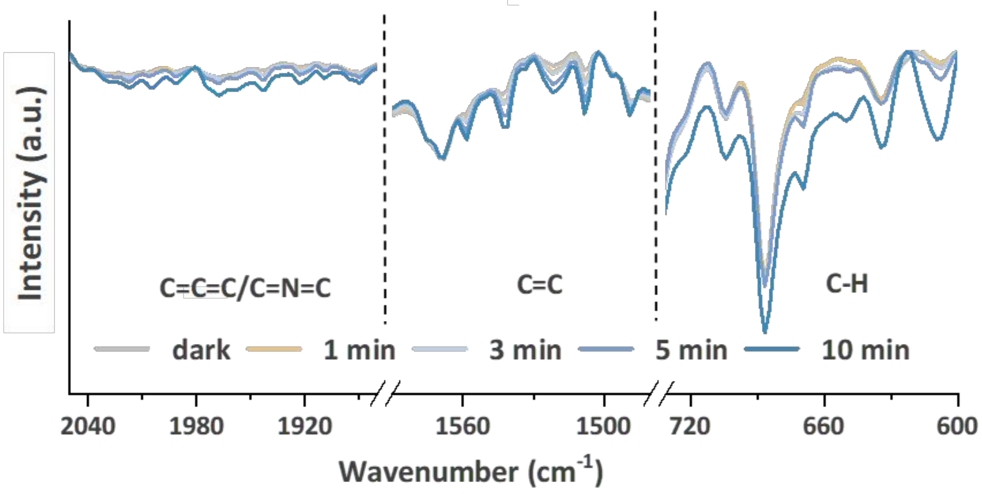

Figure S9. The FTIR spectra of the BT-Fl-Py polymer at dark and under light irradiation.

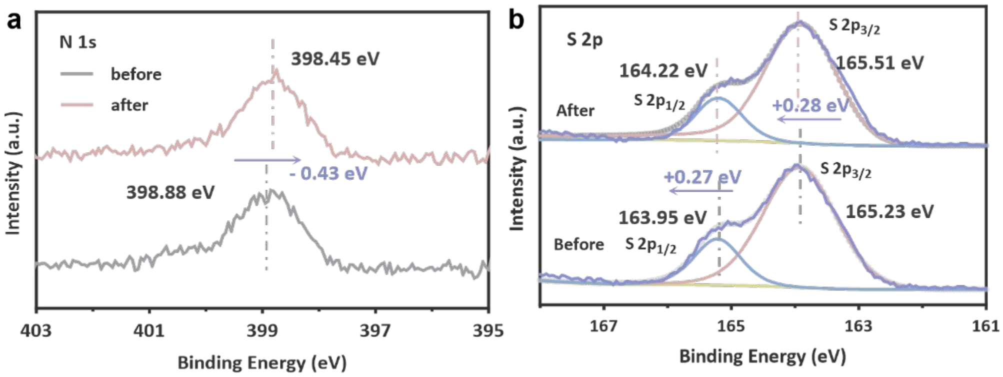

Figure S10. The XPS spectra of the BT-Fl-Py polymer before and after irradiation. (a) N 1s peak and (b) S 2p peak.

BT-Fl-Py

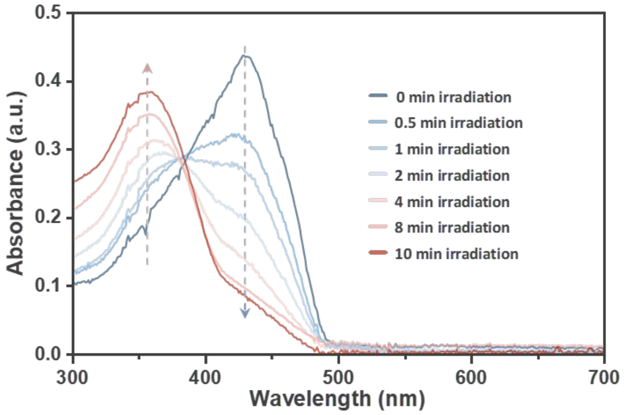

Figure S11. The UV-vis absorption spectra of BT-Fl-Py in THF before and after irradiation for different periods of time (λ_{ex} = 420 nm).

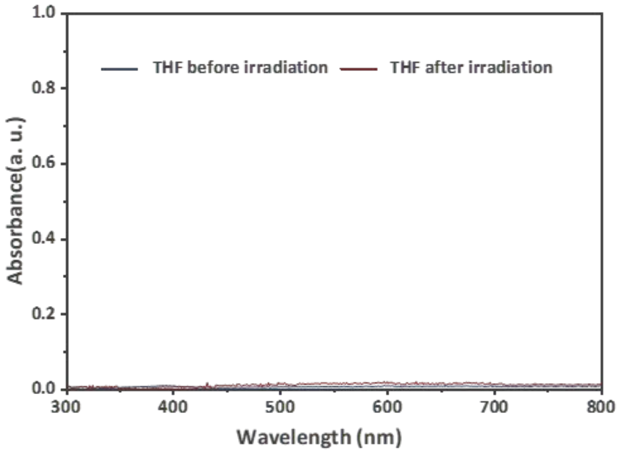

Figure S12. The UV-vis absorption spectra of the THF solvent before and after 30 min irradiation (λ_{ex} = 420 nm).

Research on the influencing factors of light-induced charge separation states

(1) Solvent control experiments.

To assess the potential role of specific solvent-solute interactions in the observed spectral evolution, control experiments were carried out in alternative polar aprotic solvents, namely N,N-dimethylformamide (DMF) and dimethyl sulfoxide (DMSO). As displayed in **Figure S13**, the photoinduced spectral changes of BT-Fl-Py in both DMF and DMSO closely match those obtained in THF, including the growth of the same photogenerated absorption features at 360 nm. The consistent spectral response across solvents with distinct chemical properties and polarities firmly establishes that the phenomenon is an intrinsic photophysical property of the material, rather than an artifact arising from specific interactions with any particular solvent.

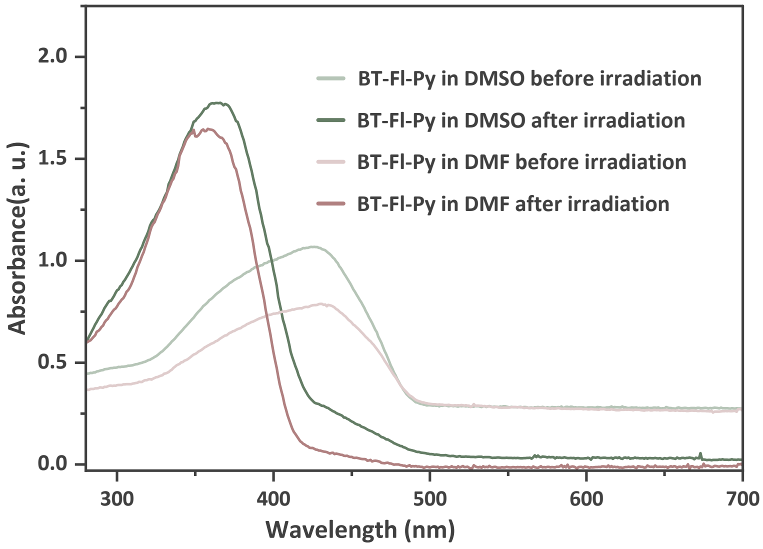

Figure S13. The UV-vis absorption spectrum of BT-Fl-Py in different solution after irradiation.

(2) Concentration control experiments.

To evaluate the potential contribution of interchain interactions to the observed spectral evolution, a concentration-dependent study was performed. As illustrated in **Figure S14**, the characteristic changes in the UV-vis absorption spectra upon irradiation remained qualitatively consistent across a concentration range from dilute to moderately concentrated solutions (10~40 mg L^{-1}). These results collectively indicate that the photophysical changes are intrinsic to the BT-Fl-Py and are not a consequence of inter-polymer chain interactions.

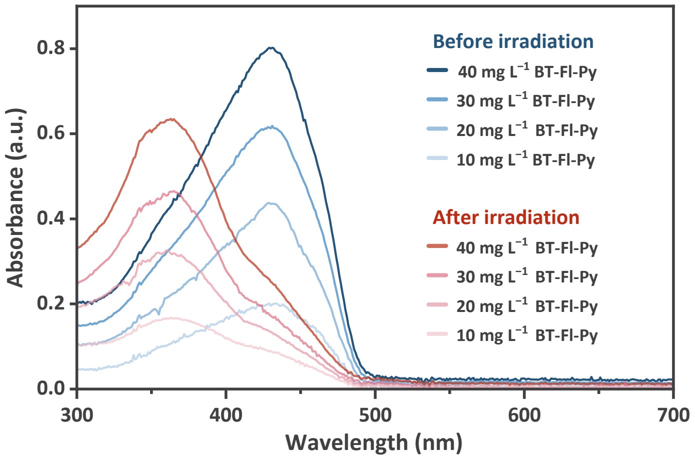

Figure S14. The UV-vis absorption spectra of BT-Fl-Py in different concentrations after irradiation.

(3) Atmosphere control experiments.

Differential absorption spectra collected after 5 min of irradiation under O_2-rich (air) versus N_2 atmosphere show a significantly larger positive feature at 360 nm in air (**Figure S15**), indicating that O_2 promotes the formation of the photoinduced π-torsional distortion. As an efficient electron scavenger, O_2 scavenges photogenerated electrons from the $TICT_1$ state, suppressing electron-hole recombination and facilitating hole accumulation on the polymer backbone. The accumulated holes reduce the barrier to conformational twisting of the fluorene π-spacer and stabilize the π-twisted state, which is reflected by the enhanced 360 nm differential absorbance under O_2-rich conditions.

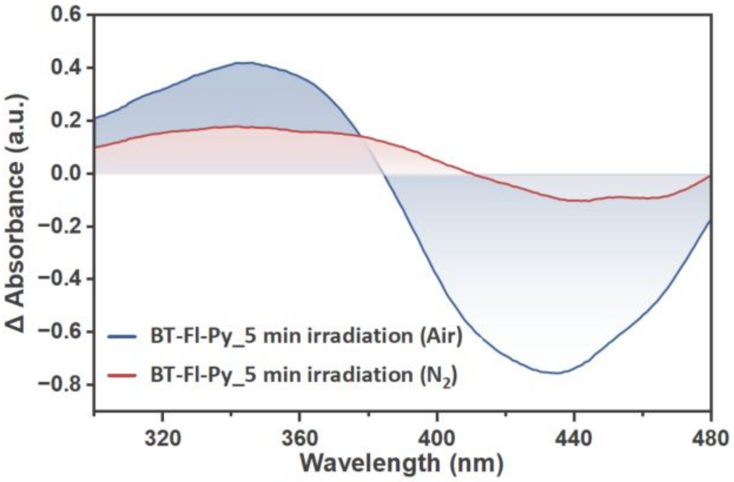

Figure S15. The effect of atmosphere on light absorption.

Reversibility of the charge separation state induced by light excitation

The recovery of the UV-vis spectral changes required a 30-min of dark storage. Specifically, the intensity of the new 360 nm absorption peak decreased, while the intensity of the ~430 nm absorption peak showed a corresponding recovery. This spectral evolution directly reflected the gradual decay of the photoinduced species and the restoration of the material to its initial ground state.

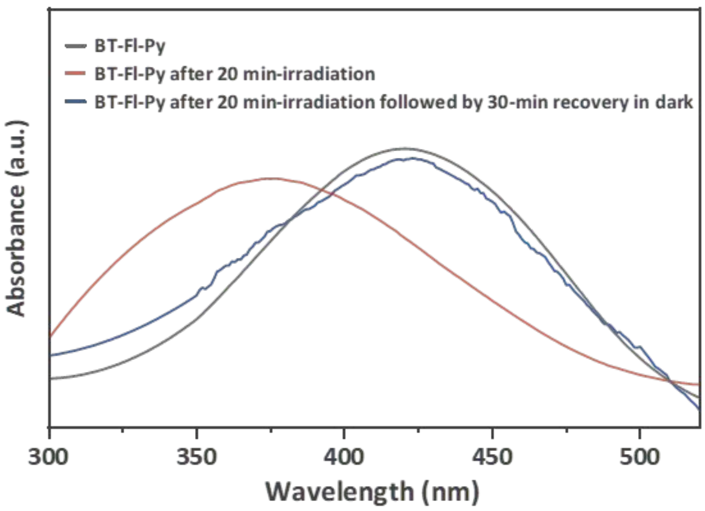

Figure S16. UV-vis absorption spectra of the BT-Fl-Py after irradiation and recovery treatment.

BT-Fl

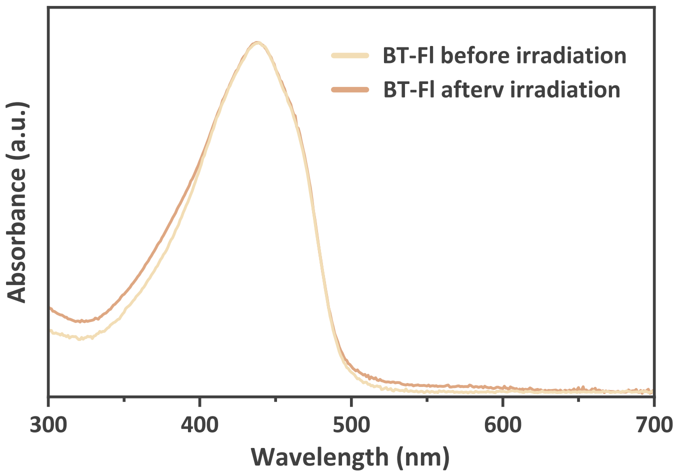

Figure S17. The UV-vis absorption spectra of BT-Fl in THF before and after irradiation. (λ_{ex} = 420 nm).

Fl

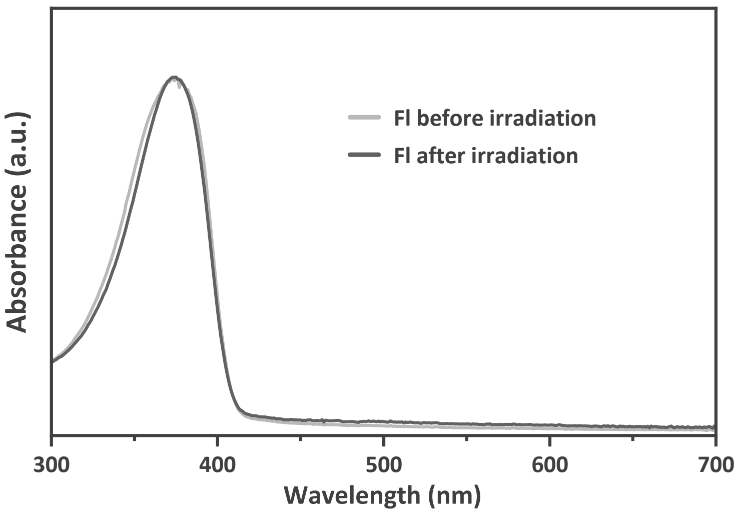

Figure S18. The UV-vis absorption spectra of Fl in THF before and after irradiation (λ_{ex} = 420 nm).

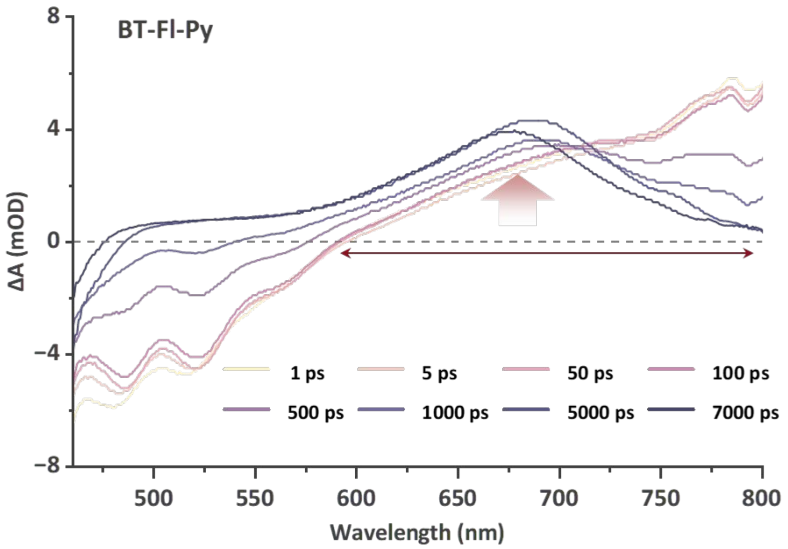

Figure S19. Femtosecond transient absorption spectra (fs-TAS) of BT-Fl-Py in THF. (λ_{ex} = 420 nm).

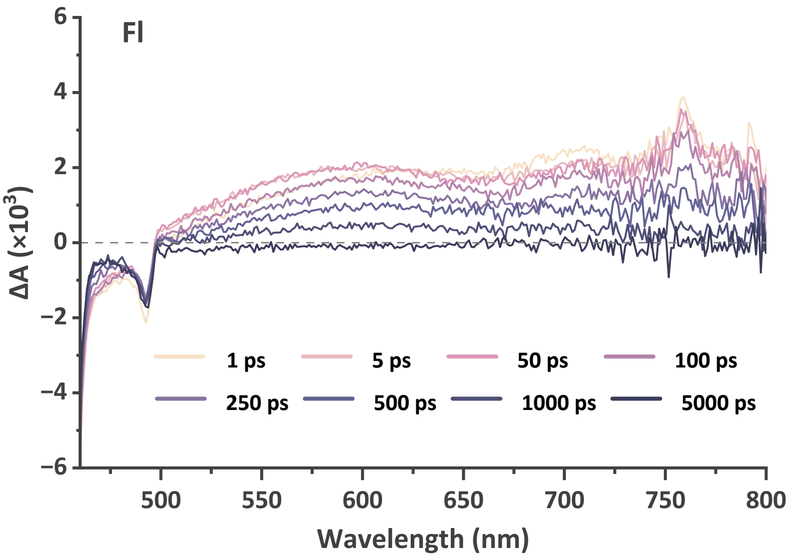

Figure S20. fs-TAS of Fl in THF. (λ_{ex} = 420 nm).

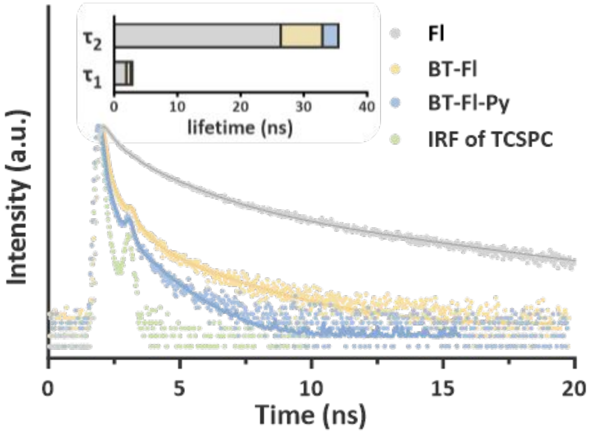

Figure S21. Instrument response function (IRF) of the TCSPC system and nanosecond-scale TRPL decay curves of Fl, BT-Fl, and BT-Fl-Py polymers (λ_{ex} = 405 nm). Measurement conditions: ambient air atmosphere, room temperature, time window = 0~20 ns. This measurement targets the short-lived prompt fluorescence component of the polymers.

BT-Fl-Py: τ_1=0.24±0.06 (A_1=1.01); τ_2=2.61±0.11 (A_2=2.63).

BT-Fl: τ_1=0.78±0.88 (A_1=1.48); τ_2=6.08±0.52 (A_2=1.93).

Fl: τ_1=2.08±0.22 (A_1=0.83); τ_2=26.53±0.51 (A_2=2.89).

Excitation analysis

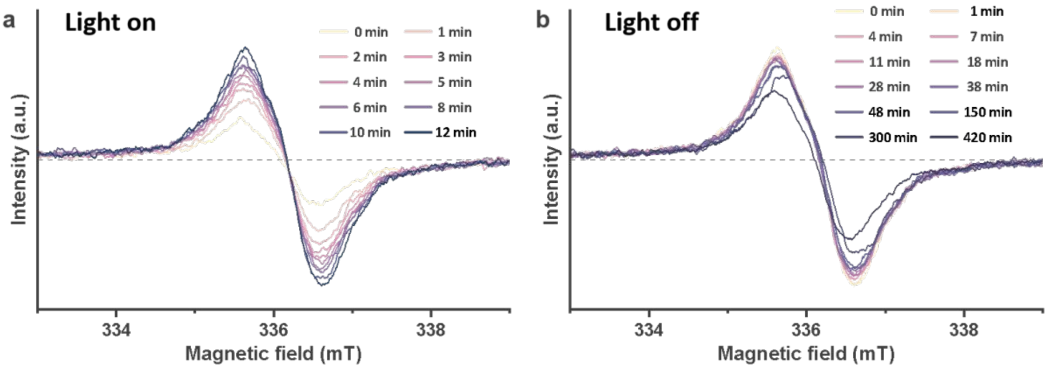

Figure S22. The *in-situ* EPR spectra of BT-Fl-Py that vary with (a) irradiation on duration and (b) irradiation off duration.

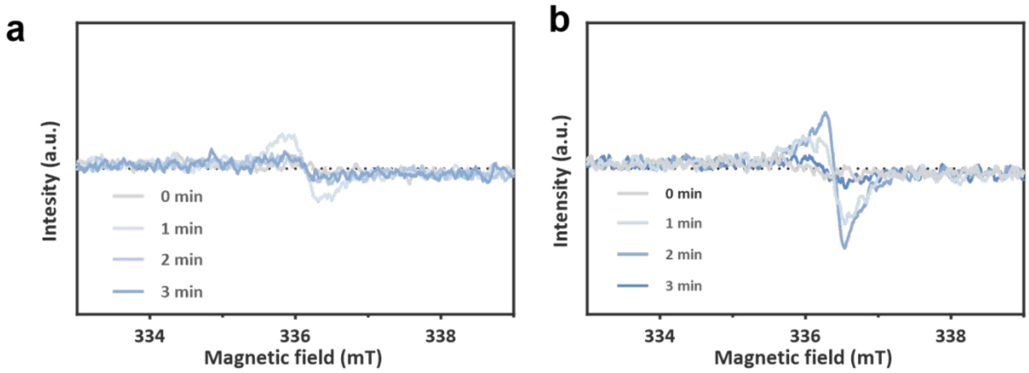

Figure S23. The *in-situ* EPR spectra of (a) Fl and (b)BT-Fl that vary with light irradiation duration.

Characterization of energy band structure

The band gap was characterized by UV-vis absorption, and the conduction band position of the photocatalyst was obtained Mott-Schottky measurement.

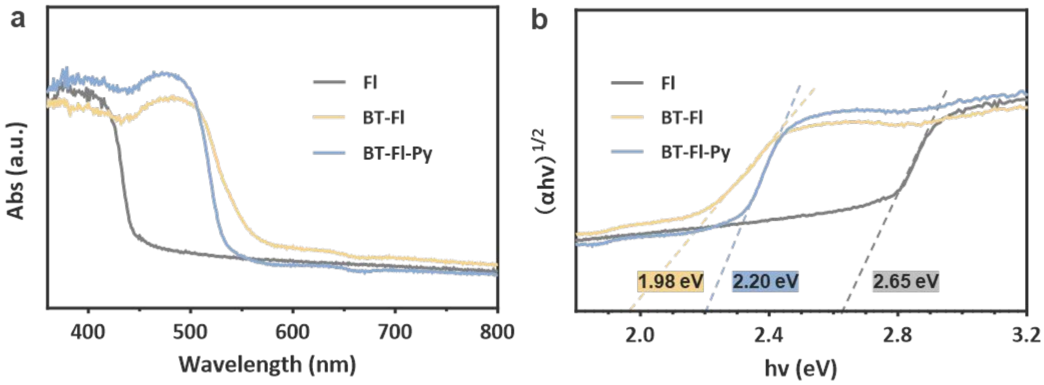

Figure S24. UV-vis DRS spectra and the corresponding band gaps. (a) UV-vis DRS spectra. (b)Tauc plot of Fl, BT-Fl and BT-Fl-Py polymers.

The band gap was determined by UV-vis absorption based on the Tauc plot:

$$(\alpha hv)^{1/2}=A(hv-E_g)$$

where α denotes the absorption coefficient, hv represents the photon energy, E_g is the bandgap, and A is constants. It can be seen from **Figure S24b** that the band gaps of Fl, BT-Fl and BT-Fl-Py were determined to be 2.65, 1.98 and 2.20 eV.

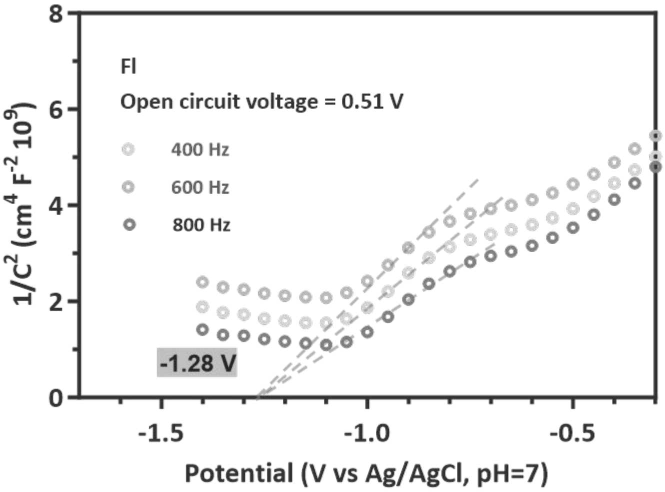

Figure S25. Mott-Schottky plots of Fl in a 0.1 M Na_2SO_4 solution.

Flat band position of Fl was obtained through Mott-Schottky plots as shown in **Figure S25**. For n-type semiconductors, the conduction band position is approximately 0.2 V more negative than the flat band potential. The flat-band potential of Fl was determined to be −1.28 V vs. Ag/AgCl, pH = 7. Considering the reduction potential of Ag/AgCl = 0.197 V and the conduction band potential was determined to be −1.34 V vs. SCE.

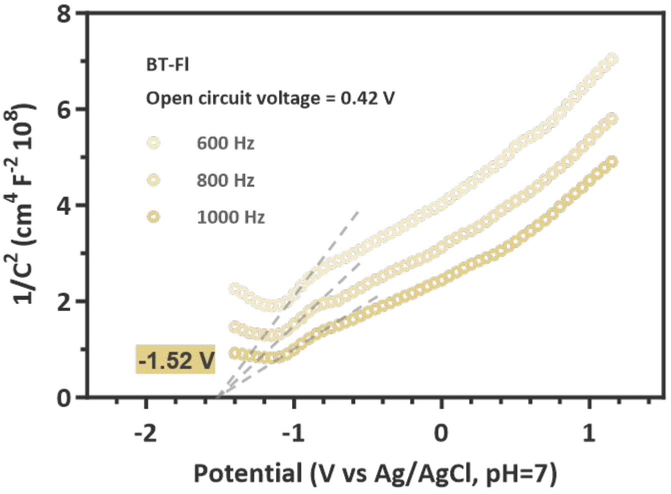

Figure S26. Mott-Schottky plots of BT-Fl in a 0.1 M Na_2SO_4 solution.

Flat band position of BT-Fl was obtained through Mott-Schottky plots as shown in **Figure S26**. For n-type semiconductors, the conduction band position is approximately 0.2 V more negative than the flat band potential. The flat-band potentials of BT-Fl was determined to be −1.56 V vs. Ag/AgCl, pH = 7. Considering the reduction potential of Ag/AgCl = 0.197 V and the conduction band potential was determined to −1.72 V vs. SCE.

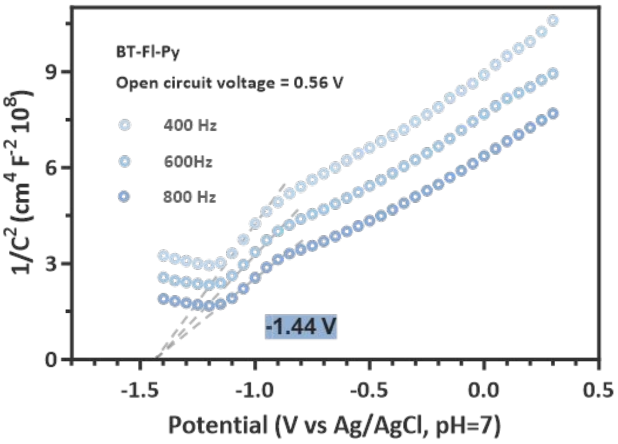

Figure S27. Mott-Schottky plots of BT-Fl-Py in a 0.1 M Na_2SO_4 solution.

Flat band position was obtained through Mott-Schottky plots as shown in **Figure S27**. For n-type semiconductors, the conduction band position is approximately 0.2 V more negative than the flat band potential. The flat-band potential of BT-Fl-Py was determined to be −1.44 V vs. Ag/AgCl, pH = 7. Considering the reduction potential of Ag/AgCl = 0.197 V and the conduction band potential was determined to be −1.60 V vs. SCE.

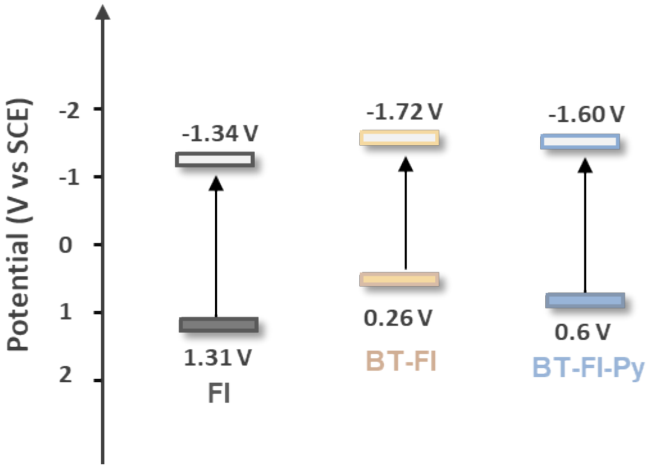

Figure S28. The energy band position of Fl, BT-Fl and BT-Fl-Py polymers.

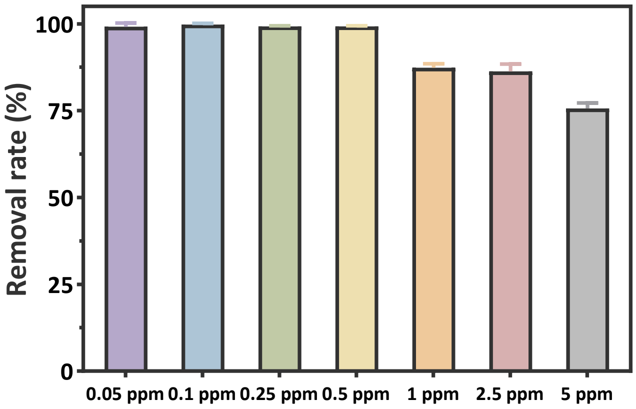

Figure S29. PFOA removal rate over BT-Fl-Py in 5 min at different PFOA concentrations.

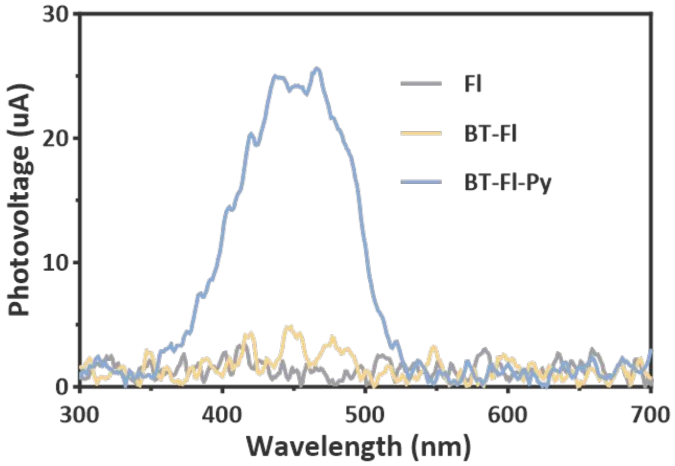

Figure S30. Surface photovoltage profiles of Fl, BT-Fl and BT-Fl-Py.

The surface photovoltage of BT-Fl-Py is significantly enhanced than that of Fl and BT-Fl, indicating improved photogenerated charge carrier separation and migration in BT-Fl-Py.

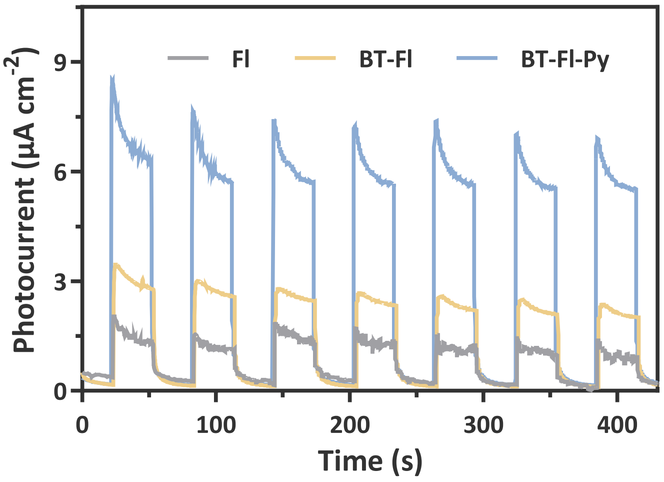

Figure S31. The photocurrent response curve of Fl, BT-Fl and BT-Fl-Py.

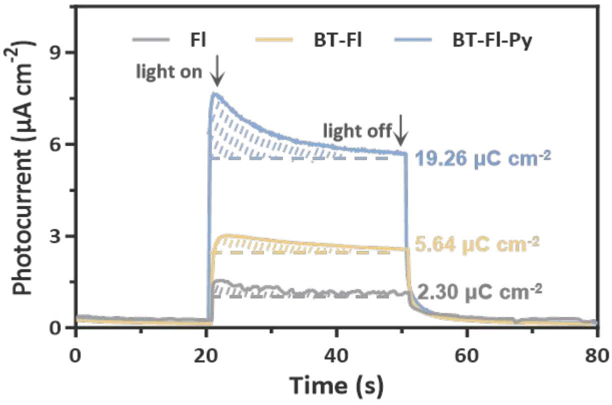

Figure S32. The surface charge density of Fl, BT-Fl and BT-Fl-Py.

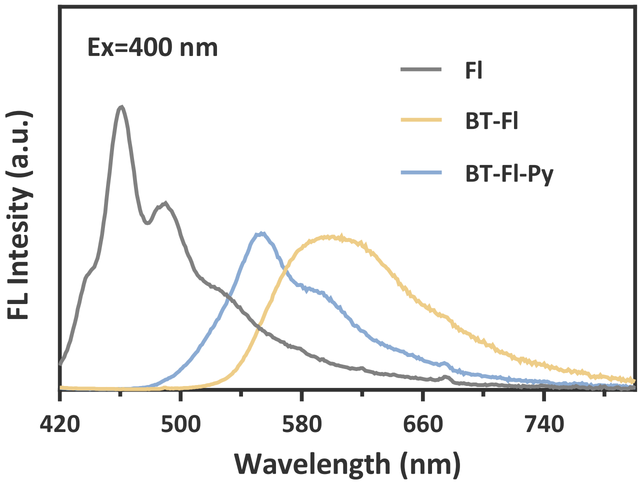

Figure S33. The steady-state PL spectra of Fl, BT-Fl and BT-Fl-Py.

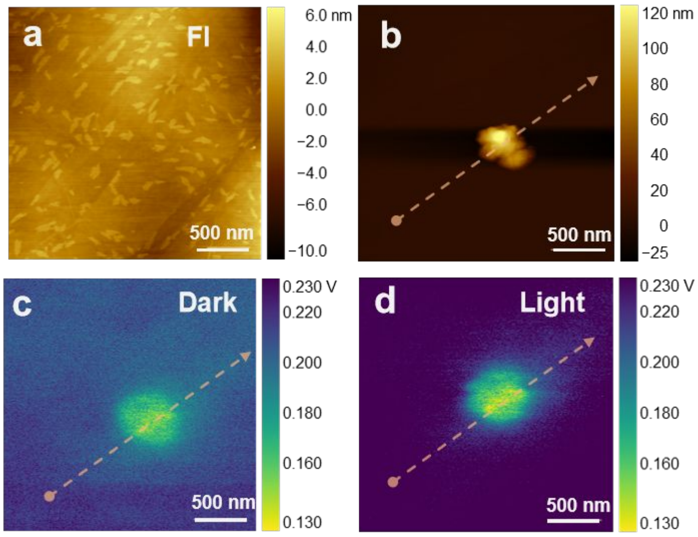

Figure S34. (a-b) The AFM images of Fl. (c-d) Surface potentials of Fl measured with KPFM before and after irradiation.

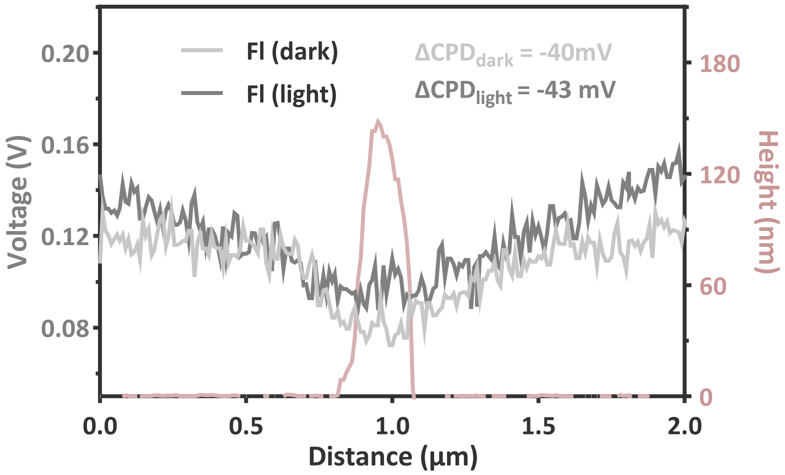

Figure S35. Contact potential difference (CPD) diagram and the sample height of Fl.

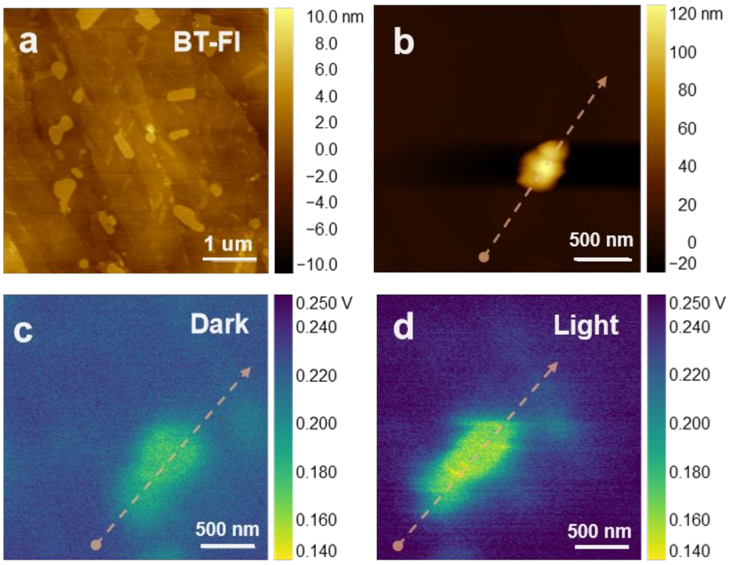

Figure S36. (a-b) The AFM images of BT-Fl. (c-d) Surface potentials of BT-Fl measured with KPFM before and after irradiation.

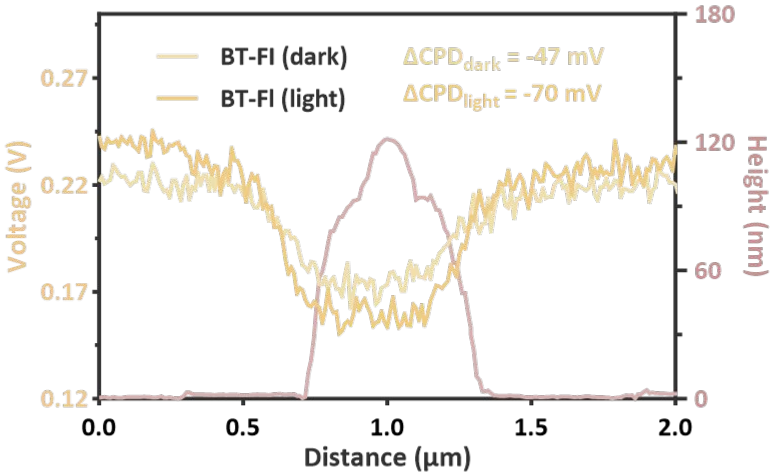

Figure S37. CPD diagram and the sample height of BT-Fl.

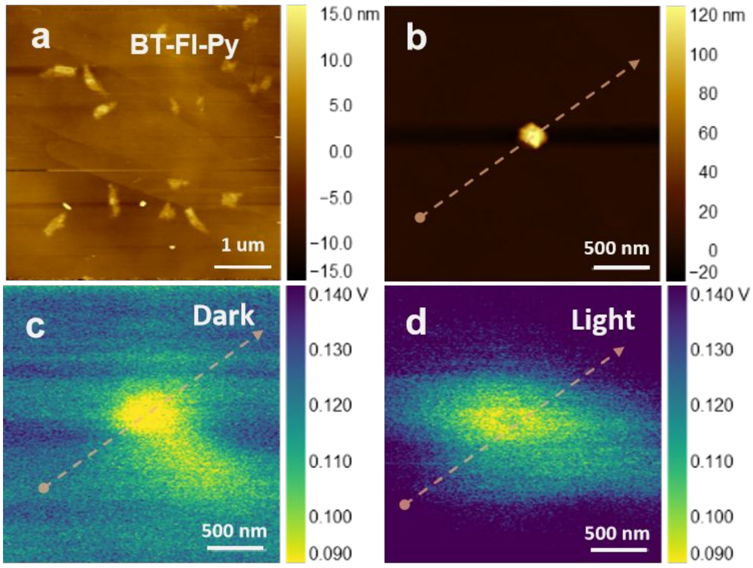

Figure S38. (a-b) The AFM images of BT-Fl-Py. (c-d) Surface potentials of BT-Fl-Py measured with KPFM before and after irradiation.

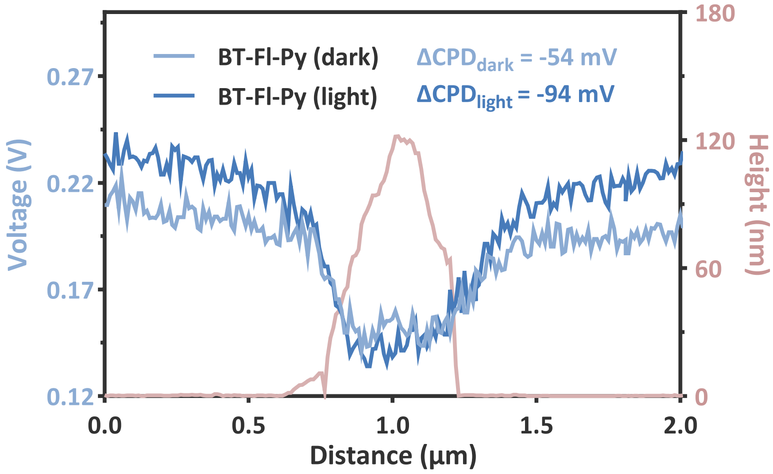

Figure S39. CPD diagram and the sample height of BT-Fl-Py.

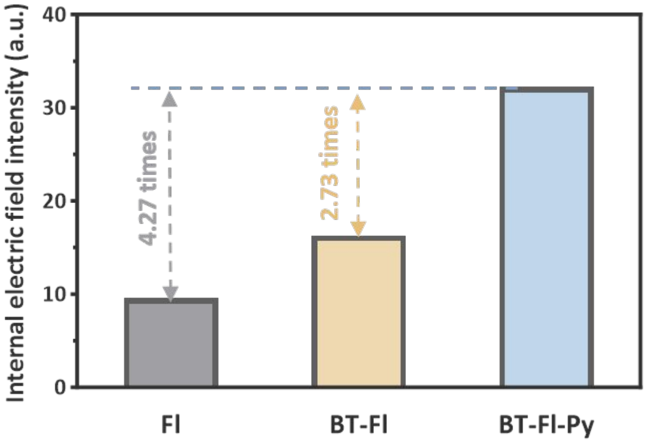

Figure S40. Internal electric field intensities of Fl, BT-Fl and BT-Fl-Py.

Figure S40 showed that BT-Fl-Py displayed a stronger built-in electric field, 4.27 and 2.73 times higher than that of Fl and BT-Fl, indicating significantly promoted charge separation and transfer in BT-Fl-Py.

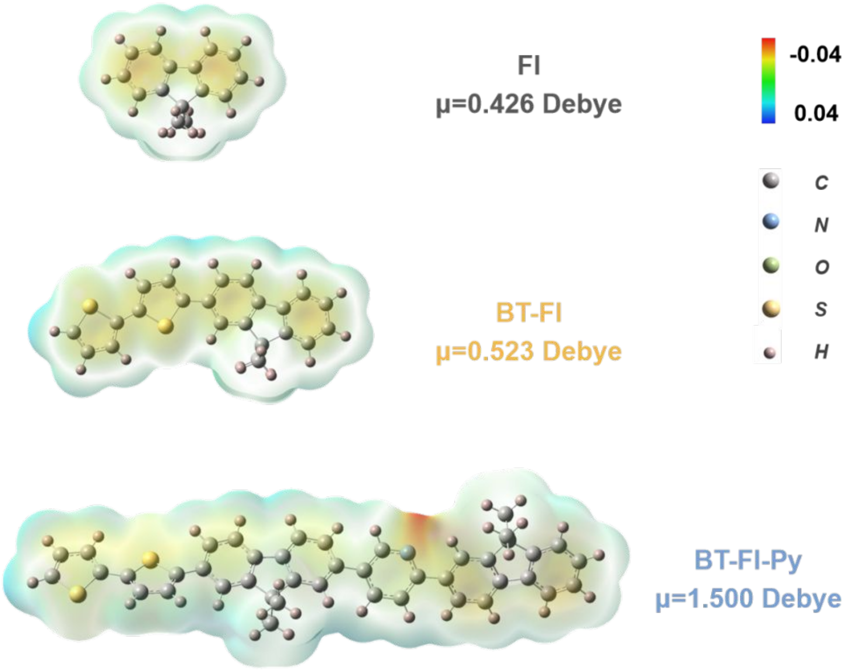

Figure S41. Electrostatic potential (ESP) distribution map and molecular dipole of Fl, BT-Fl and BT-Fl-Py.

The ESP distribution maps and molecular dipoles showed a substantial elevation in molecular dipole, escalating from 0.426 Debye for Fl to 0.523 Debye for BT-Fl to 2.513 Debye for BT-Fl-Py, benefited from the directional charge difference formed with the bithiophene as donor and pyridine as acceptor.

Defluorination experiment for PFOA

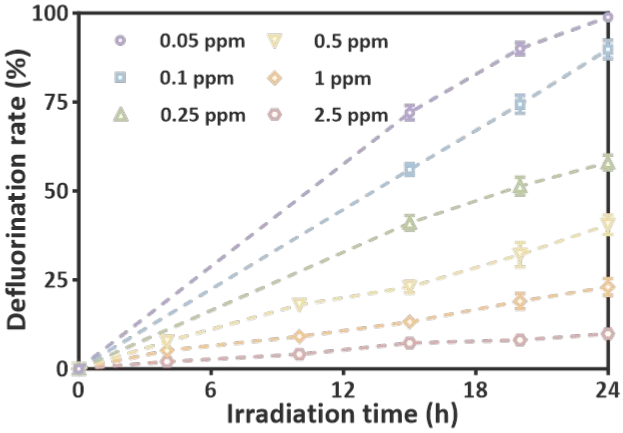

Figure S42. Defluorination kinetics curves for PFOA at different concentrations (λ = 360~780 nm).

Stability of the catalyst

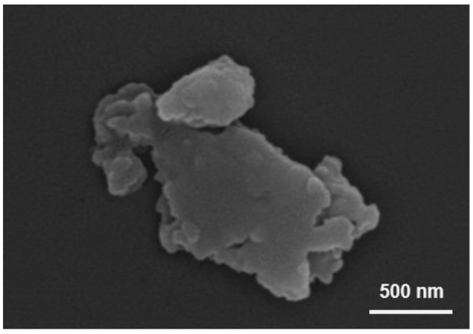

Figure S43. The SEM image of the BT-Fl-Py polymer after three cycles of photocatalytic experiments.

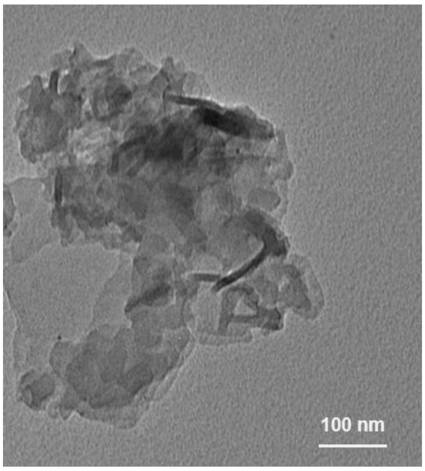

Figure S44. The TEM image of the BT-Fl-Py polymer after three cycles of photocatalytic experiments.

The physical and chemical properties of photocatalysts

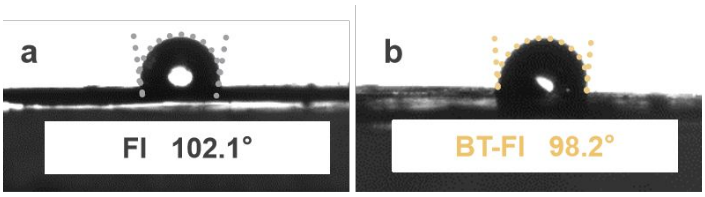

Figure S45. The water contact angle of (a) Fl and (b) BT-Fl.

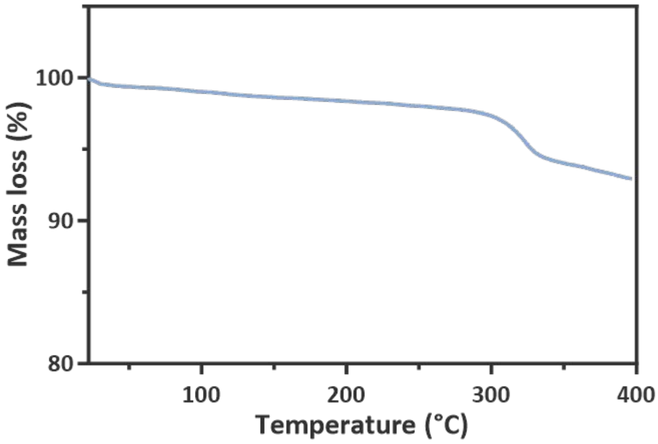

Figure S46. The thermogravimetric (TGA) analysis of BT-Fl-Py polymer.

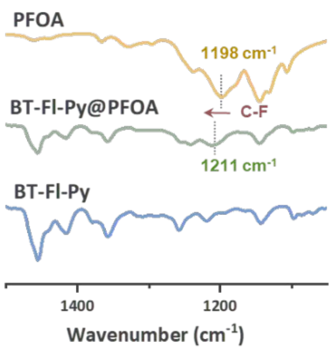

Figure S47. FT-IR spectra showing adsorption-induced vibrational shifts.

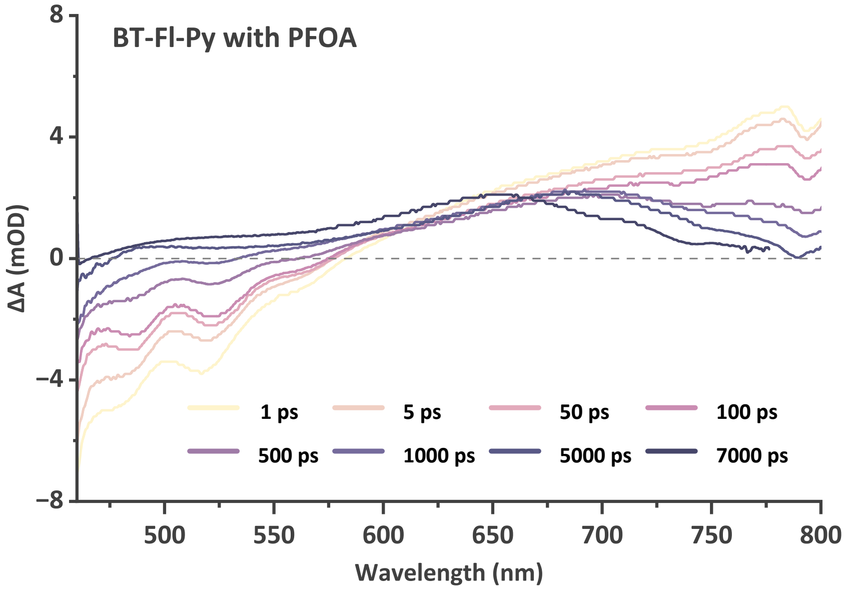

Figure S48. fs-TAS of BT-Fl-Py with PFOA in THF. (λ_{ex} = 420 nm).

Identification of reaction intermediates

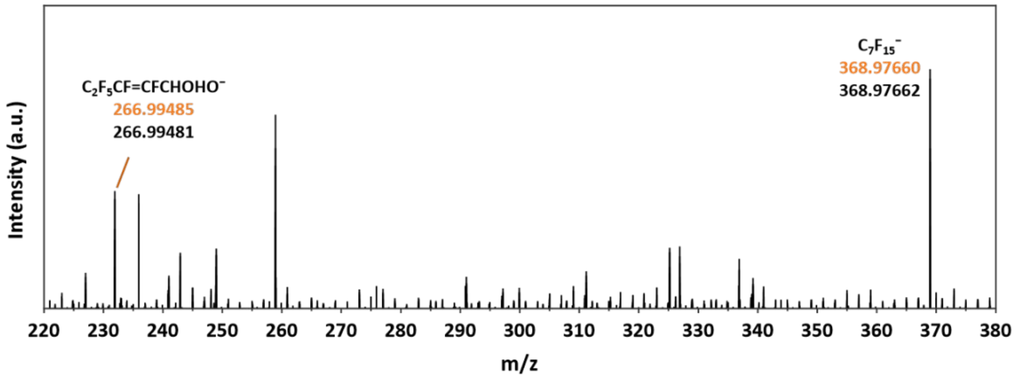

Figure S49. ESI-MS spectrum of the 0.2 ppm PFOA degradation products for 24 h (λ = 360~780 nm).

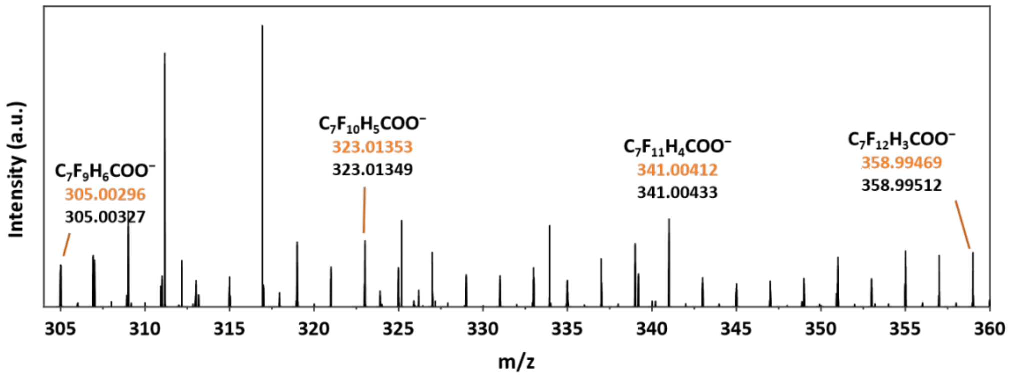

Figure S50. ESI-MS spectrum of the 0.2 ppm PFOA degradation products for 24 h (λ = 360~780 nm).

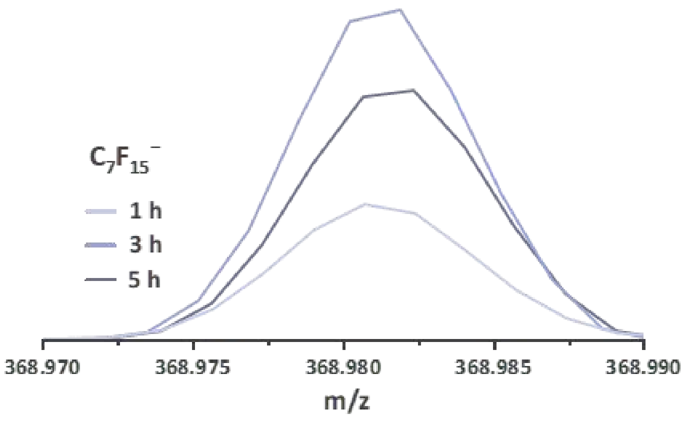

Figure S51. The $C_7F_{15}^-$ detected by LC-MS at different photocatalytic reaction times. ($C_7F_{15}^-$, exact mass: 368.978 m/z, found: 368.981 m/z).

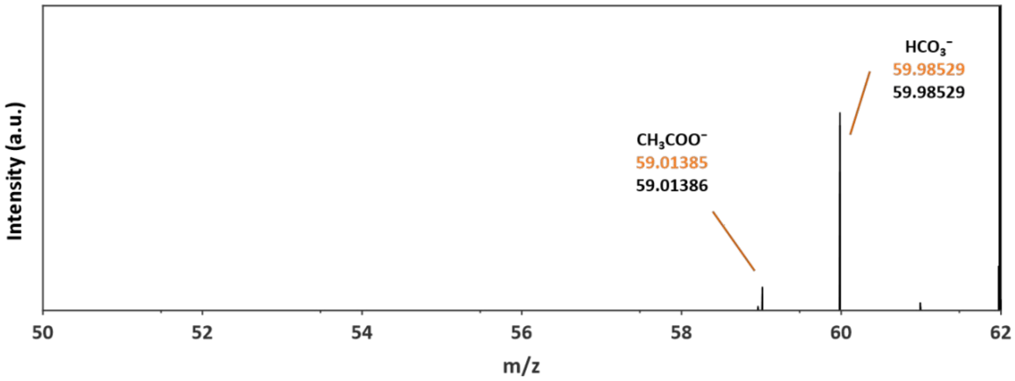

Figure S52. ESI-MS spectrum of the 0.05 ppm PFOA degradation products for 24 h (λ = 360~780 nm).

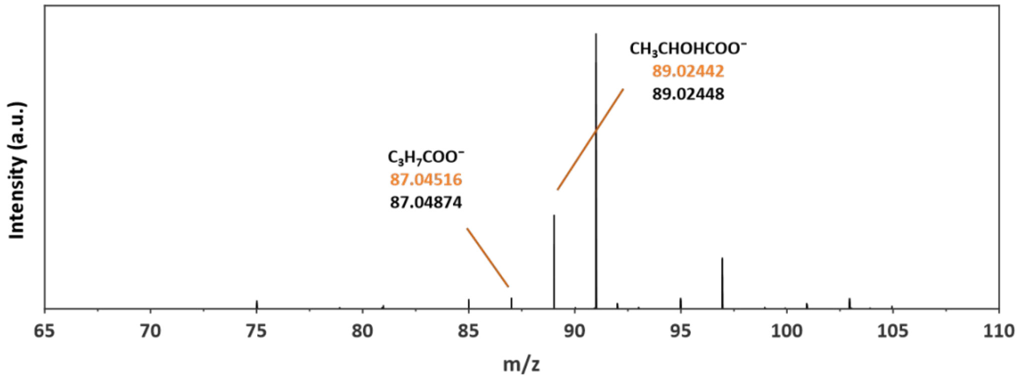

Figure S53. ESI-MS spectrum of the 0.05 ppm PFOA degradation products for 24 h (λ = 360~780 nm).

Table S1. Theoretical calculation results of the single-point energy and adsorption energy of BT-Fl-Py and $PFOA^-$.

**BT-Fl-Py (kJ/mol)**	**$PFOA^-$ (kJ/mol)**	**BT-Fl-Py@ $PFOA^-$ (kJ/mol)**	**ΔG_{ads} (kJ/mol)**
−6589764.8350	−5127713.3988	−11717586.2679	−108.034

Table S2. Anion captured by LC-MS.

Anion	Exact Mass (m/z)	Found (m/z)
$C_7F_{15}^-$	368.97660	368.97684
$C_2F_5CF{=}CFCHOHO^-$	226.99485	226.99495
$C_7F_{12}H_3COO^-$	358.99469	358.99512
$C_7F_{11}H_4COO^-$	341.00412	341.00433
$C_7F_{10}H_5COO^-$	323.01353	323.01349
$C_7F_9H_6COO^-$	305.00296	305.00327
CH_3COO^-	59.01385	59.01386
$C_3H_7COO^-$	87.04516	87.04817
$CH_3CHOHCOO^-$	89.02442	89.02448
HCO_3^-	59.98528	59.98529

References

[1] Chen, J., Dong, C.-L., Zhao, D., Huang, Y.-C., Wang, X., Samad, L., Dang, L., Shearer, M., Shen, S., Guo, L., Molecular design of polymer heterojunctions for efficient solar-hydrogen conversion. *Adv. Mater.* **2017,** *29* (21), 1606198.

[2] Han, C., Xiang, S., Xie, P., Dong, P., Shu, C., Zhang, C., Jiang, J. X., A universal strategy for boosting hydrogen evolution activity of polymer photocatalysts under visible light by inserting a narrow-band-gap spacer between donor and acceptor. *Adv. Funct. Mater.* **2022,** *32* (16).

[3] Frisch, M. J., Trucks, G. W., Schlegel, H. B., Scuseria, G. E., Robb, M. A., Cheeseman, J. R., Scalmani, G., Barone, V., Petersson, G. A., Nakatsuji, H., Li, X., Caricato, M., Marenich, A. V., Bloino, J., Janesko, B. G., Gomperts, R., Mennucci, B., Hratchian, H. P., Ortiz, J. V., Izmaylov, A. F., Sonnenberg, J. L., Williams, Ding, F., Lipparini, F., Egidi, F., Goings, J., Peng, B., Petrone, A., Henderson, T., Ranasinghe, D., Zakrzewski, V. G., Gao, J., Rega, N., Zheng, G., Liang, W., Hada, M., Ehara, M., Toyota, K., Fukuda, R., Hasegawa, J., Ishida, M., Nakajima, T., Honda, Y., Kitao, O., Nakai, H., Vreven, T., Throssell, K., Montgomery Jr., J. A., Peralta, J. E., Ogliaro, F., Bearpark, M. J., Heyd, J. J., Brothers, E. N., Kudin, K. N., Staroverov, V. N., Keith, T. A., Kobayashi, R., Normand, J., Raghavachari, K., Rendell, A. P., Burant, J. C., Iyengar, S. S., Tomasi, J., Cossi, M., Millam, J. M., Klene, M., Adamo, C., Cammi, R., Ochterski, J. W., Martin, R. L., Morokuma, K., Farkas, O., Foresman, J. B., Fox, D. J. *Gaussian 16 Rev. C.01*, Wallingford, CT, 2016.

[4] Lu, T., Chen, Q., Independent gradient model based on Hirshfeld partition: A new method for visual study of interactions in chemical systems. *J. Comput. Chem.* **2022,** *43* (8), 539-555.

[5] Lu, T., Visualization analysis of covalent and noncovalent interactions in real space. *Angew. Chem. Int. Ed.* **2025,** *64* (29), e202504895.

[6] Lu, T., A comprehensive electron wavefunction analysis toolbox for chemists, Multiwfn. *J. Chem. Phys.* **2024,** *161* (8).

[7] Lu, T., Chen, F., Multiwfn: A multifunctional wavefunction analyzer. *J. Comput. Chem.* **2011,** *33* (5), 580-592.

[8] William Humphrey, Andrew Dalke, Schulten, K., VMD: visual molecular dynamics. *J. Mol. Graph.* **1996,** *14*.

[9] Takashi Kanata, M. M., Hideyuki Takakura, and Yoshihiro Hamakawa, Photoreflectance characterization of built-in potential in MBE produced as-grown GaAs surface. *Modul. Spectr.* **1990,** *1286* 56-65.

[10] Le Formal, F., Sivula, K., Grätzel, M., The transient photocurrent and photovoltage behavior of a hematite photoanode under working conditions and the influence of surface treatments. *J. Phys. Chem. C* **2012,** *116* (51), 26707-26720.
